# Changes in Tryptophan-Kynurenine Metabolism in Patients with Depression Undergoing ECT—A Systematic Review

**DOI:** 10.3390/ph15111439

**Published:** 2022-11-19

**Authors:** Tore Ivar Malmei Aarsland, Johanne Telnes Instanes, Maj-Britt Rocio Posserud, Arve Ulvik, Ute Kessler, Jan Haavik

**Affiliations:** 1 Department of Biomedicine, University of Bergen, 5020 Bergen, Norway; 2 Department of Clinical Medicine, University of Bergen, 5020 Bergen, Norway; 3 Division of Psychiatry, Haukeland University Hospital, 5021 Bergen, Norway; 4 Bevital A/S, Laboratoriebygget, 5020 Bergen, Norway

**Keywords:** electroconvulsive therapy, depression, tryptophan, kynurenine, quinolinic acid, inflammation, kidney function, stress, age, comorbidity

## Abstract

The kynurenine pathway of tryptophan (Trp) metabolism generates multiple biologically active metabolites (kynurenines) that have been implicated in neuropsychiatric disorders. It has been suggested that modulation of kynurenine metabolism could be involved in the therapeutic effect of electroconvulsive therapy (ECT). We performed a systematic review with aims of summarizing changes in Trp and/or kynurenines after ECT and assessing methodological issues. The inclusion criterium was measures of Trp and/or kynurenines before and after ECT. Animal studies and studies using Trp administration or Trp depletion were excluded. Embase, MEDLINE, PsycInfo and PubMed were searched, most recently in July 2022. Outcomes were levels of Trp, kynurenines and ratios before and after ECT. Data on factors affecting Trp metabolism and ECT were collected for interpretation and discussion of the reported changes. We included 17 studies with repeated measures for a total of 386 patients and 27 controls. Synthesis using vote counting based on the direction of effect found no evidence of effect of ECT on any outcome variable. There were considerable variations in design, patient characteristics and reported items. We suggest that future studies should include larger samples, assess important covariates and determine between- and within-subject variability. PROSPERO (CRD42020187003).

## 1. Introduction

**Figure 1:**
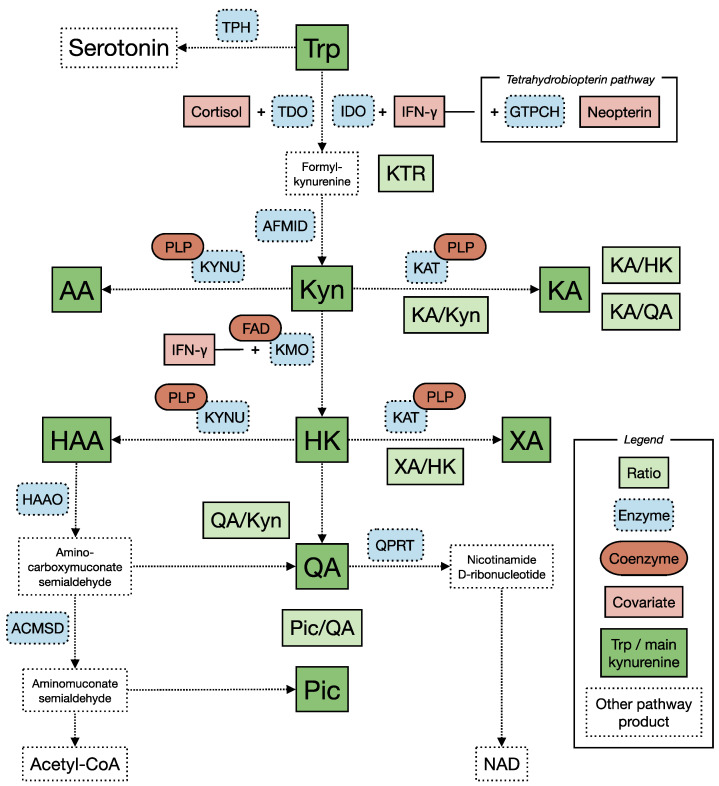
The kynurenine pathway of Trp metabolism. Abbreviations: Acetyl-CoA, acetyl coenzyme A; ACMSD, aminocarboxymuconate semialdehyde decarboxylase; AA, anthranilic acid; AFMID, arylformamidase; FAD, flavin adenine dinucleotide; CAA, competing amino acid; GTPCH, GTP cyclohydrolase; HAA, 3-hydroxyanthranilic acid; HAAO, 3-hydroxyanthranilate 3,4-dioxygenase; HK, 3-hydroxykynurenine; IDO, indoleamine 2,3-dioxygenase; KA, kynurenic acid; KAT, kynurenine aminotransferase; KMO, kynurenine monooxygenase; KTR, kynurenine-tryptophan ratio; Kyn, kynurenine; KYNU, kynureninase; LAT1, L-type amino acid transporter; NEFA, non-esterified fatty acid; NAD, nicotinamide adenine dinucleotide; Pic, picolinic acid; PLP, pyridoxal 5′-phosphate;QA, quinolinic acid; QPRT, quinolinate phosphoribosyltransferase; TDO, tryptophan 2,3-dioxygenase; IFN-γ, interferon gamma; Trp, tryptophan; TPH, tryptophan hydroxylase; XA, xanthurenic acid.

An increasing number of cross-sectional studies, intervention studies and meta-analyses suggest that the kynurenine pathway of tryptophan (Trp) metabolism (Figure 1) is involved in the pathophysiology of depression and other psychiatric disorders (reviewed in [1,2,3,4,5,6]). The kynurenine pathway includes multiple metabolites (collectively known as kynurenines), several of which have important properties related to cellular energy (reviewed in [7,8]), glutamatergic signaling (reviewed in [9]), regulation of immune system activity (reviewed in [10]) and production and scavenging of reactive oxygen species (reviewed in [11]). Correspondingly, the kynurenine pathway has been implicated in a wide range of somatic and psychiatric conditions (reviewed in [2,12,13,14]).

Two main hypotheses link the Trp-kynurenine metabolism to depression (reviewed in [15]). The original hypothesis focuses on brain serotonin production, which can be limited by low levels of Trp, its essential precursor [16]. The kynurenine pathway is the main metabolic pathway for Trp [17] and is even more active under inflammatory conditions [8], which has been associated with depression [18,19,20,21]. Pathway activation is often assessed by measuring the relative concentrations of Trp and kynurenine (Kyn), the first stable metabolite of the pathway, i.e., the kynurenine-tryptophan ratio (KTR), together with levels of inflammatory markers. Depression has been hypothesized to be related to suboptimal serotonin signaling [15], and low availability of Trp for serotonin synthesis could potentially contribute to depression. Deficiency of Trp, for instance through activation of the kynurenine pathway, has therefore been investigated as a potential cause for reduced serotonin production in depression [22].

An alternative hypothesis suggests that alterations in concentrations of kynurenines themselves could play a role in depression [22,23,24,25]. Here, the most focus has been placed on the pathway’s neurotoxic potential and on three key pathway metabolites: kynurenic acid (KA), 3-hydroxykynurenine (HK) and quinolinic acid (QA). KA is a pathway branch product and an antagonist of the N-methyl-D-aspartate receptor (NMDAr) [26] that can inhibit presynaptic glutamate release and disrupt excitatory synaptic function [27]. QA exerts the opposite effect as an NMDAr agonist [26]. It is increased under inflammatory conditions and may cause neurotoxicity through multiple mechanisms [28]. Similarly, HK has been shown to have a neurotoxic potential, mainly as a free radical generator [29]. Thus, QA and HK are generally considered neurotoxic, while KA is considered neuroprotective [23]. The balance between the neuroprotective and neurotoxic effects of kynurenine pathway metabolites has been considered in many studies of depression, by analyzing levels of single metabolites and ratios such as KA to QA (KA/QA) and KA/HK. Several meta-analyses have shown that patients with major depressive disorder and bipolar depression have reduced neuroprotection compared to non-depressed individuals, with comparatively lower levels of KA and lower KA/HK and KA/QA in the blood [1,4,30,31]. Therapeutic interventions have been postulated to normalize these metabolite levels and restore a balance between their neuroactive effects [32].

Electroconvulsive therapy (ECT) is an effective treatment option for severe or treatment resistant depressive episodes, with a relatively rapid onset of effect compared to pharmacological therapy. The exact therapeutic mechanisms of action are still unknown, and several studies have been performed to investigate the possible role of Trp and the kynurenine pathway, including measures related to Trp availability and kynurenine pathway activation, and the balance between neuroactive metabolites. While some studies have reported an improved neuroprotective balance after ECT [33,34], other studies have not been able to replicate these findings [35,36,37]. The reasons for this inconsistency are unclear. There are multiple methodological challenges when analyzing changes in Trp metabolism in relation to ECT. These include potential intermediary or confounding effects from a wide range of factors such as inflammation, diet, medication and somatic disease [14,38,39].

In this systematic review, we wanted to summarize available results from studies on ECT and Trp-kynurenine metabolism and to use this as context for a discussion of methodology and the way forward. The aims of this review were to:

(1) summarize the findings of studies on changes in concentrations of Trp and kynurenines after ECT;
(2) review important factors that could potentially affect the analyses of these metabolites in relation to therapeutic outcome;
(3) consider the clinical role of measures of Trp and kynurenines;
(4) propose how future studies should be designed to meet methodological issues and clarify the role of Trp metabolism in ECT.

## 2. Methods

### 2.1. Literature Search and Study Selection

#### 2.1.1. Protocol

This systematic review was conducted following the guidelines presented in the Preferred Reporting Items for Systematic Reviews and Meta-Analyses (PRISMA) statement [40] and in Synthesis Without Meta-Analysis (SWiM) [41]. A review protocol was submitted to the international prospective register of systematic reviews (PROSPERO) (https://www.crd.york.ac.uk/prospero/) accessed on 5 May 2020, and published on 5 July 2020 (registration number CRD42020187003).

#### 2.1.2. Eligibility Criteria

The inclusion criterium for this review was measurements of Trp and/or kynurenines before and after ECT or description of change in these markers after ECT. There were no restrictions on participant characteristics, report type or language integrated in the search, except for in PubMed where animal studies were excluded. Thus, the search syntax was structured with two parts: (1) terms related to Trp and the kynurenine pathway, and (2) terms related to ECT. In the subsequent screening, the following reports were excluded: animal studies, letters, conference abstracts, case reports, commentaries or reviews and reports of studies using Trp administration or Trp depletion, unless the study also included a patient group that received ECT alone or with placebo.

#### 2.1.3. Information Sources

The systematic literature search was developed by TIMA, UK and JTI. It was conducted by TIMA on 3 June 2020, in four databases: (1) Embase 1974 to 2 June 2020, (2) Ovid MEDLINE and Epub Ahead of Print, In-Process & Other Non-Indexed Citations and Daily 1946 to 2 June 2020, (3) APA PsycInfo 1806 to May Week 4 2020 and (4) PubMed (up to 3 June 2020). The search used free text and index terms for Trp-kynurenine metabolism and ECT in titles and abstracts. See Supplementary document for full search syntax. An additional systematic literature search was conducted by TIMA on 15 June 2022, in (1) Embase 1974 to 19 July 2022, (2) Ovid MEDLINE and Epub Ahead of Print, In-Process, In-Data-Review & Other Non-Indexed Citations and Daily 1946 to 19 July 2022, (3) APA PsycInfo 1806 to July Week 2 2022 and (4) PubMed. Here, the searches were limited to years 2020–2022 for the first three databases and 4 June 2020–20 July 2022 for PubMed. In addition, reference lists in included studies were scanned for relevant studies potentially not found through the systematic literature search.

#### 2.1.4. Study Selection

All records collected from the original systematic search were screened by UK and TIMA independently, based on title and abstract. Records collected in the additional search were screened by TIMA. Studies that fulfilled one or more exclusion criteria were excluded. For remaining studies, the full text was retrieved. The full text was also retrieved for records where title and abstract did not provide enough information to determine eligibility. Full-text reports that met the inclusion criterium, and none of the exclusion criteria, were considered eligible and included in the review. Disagreement regarding study eligibility was resolved through discussion (UK, TIMA).

### 2.2. Data Collection

The data collection was performed by TIMA. Each study’s aims, inclusion and exclusion criteria, outcome variables, analyses methods, main results, discussion points and conclusions were recorded for the purpose of study presentation.

For the first review aim (changes in concentrations of Trp and kynurenines after ECT), the following data were collected as primary outcomes: measures of Trp, Kyn, KA, anthranilic acid (AA), HK, xanthurenic acid (XA), 3-hydroxyanthranilic acid (HAA), picolinic acid (Pic) and QA, and of metabolite ratios KTR, KA/Kyn, KA/HK, KA/QA, XA/HK, PA/QA and QA/Kyn. These could be concentrations at any timepoint before, during or after ECT, or measures of change from before ECT to after ECT.

To address the third aim (assessment of the clinical potential of Trp and kynurenine measures), concentrations of these markers were also collected as primary outcomes for control groups at baseline and follow up when available. Associated summary statistics and *p*-values from analyses of change were also collected. For three studies [33,34,36], concentrations of the primary outcome variables were not present in the report. For two of these studies [34,36], these data were provided by the authors after written request. For the third study, the authors were contacted but could not provide the concentrations [33].

To meet the second aim of this study (effect modifiers and confounders), a wide range of pre-selected data items that could be relevant for baseline levels and analyzes of changes in Trp and kynurenines after ECT were extracted from each of the included studies. Such factors include known determinants of blood levels of Trp and kynurenines, predictors of treatment outcome and potential confounders affecting both aspects of the treatment and the outcome measure. This aspect of the review substituted the “study risk of bias assessment” described in the PRISMA 2020 checklist, with the purpose of providing points for discussion rather than assigning weights to each study in the context of a meta-analysis. Thus, from each included study we extracted data on factors related to Trp-kynurenine metabolism (inflammation, age, kidney function, BMI, sex, nutrition, fasting, B vitamin status, stress, alcohol consumption, smoking, non-esterified fatty acids (NEFAs), large neutral amino acids/competing amino acids (CAAs), glucose, medication and liver function), patient characteristics (depression characteristics, medication and comorbidity), and intervention and study design (clinical measures, ECT details, anesthesia, sample timepoints, and evaluation of treatment response or remission).

### 2.3. Synthesis and Presentation

#### 2.3.1. Grouping

The included studies were categorized first by treatment design (series or single ECT), secondly by primary outcome (kynurenines or Trp) and thirdly by year of publication. This grouping was used for all tables and figures.

#### 2.3.2. Effect Measures

When available, unadjusted mean or median concentrations before and after ECT were used to calculate percentage change (100×([post]−[pre])/[pre]) for each primary outcome variable for each study. This was also performed for concentration changes in control groups with repeated measures. To enable direct comparison, all concentrations were presented as µmol/L and nmol/L.

#### 2.3.3. Synthesis

Vote counting based on the direction of effect is a method that can be used to check if there is any evidence of an effect [42]. This was performed using the Exact Binomial Test in RStudio (binom.test) [43]. For primary outcomes reported in five or more studies (the minimal number of outcomes necessary for finding a *p*-value below 0.05), the number of studies reporting increased concentration was divided by the total number of studies reporting this outcome variable. The test null hypothesis was that two possibilities of increase and decrease were equally likely.

#### 2.3.4. Data Presentation Methods

All levels of Trp and kynurenine pathway metabolites and ratios before and after ECT were collected in a table. The calculated percentage change in these markers after ECT for patients and controls from all included studies were gathered in a table and color coded by direction and the reported statistical significance. Baseline concentrations of the collected primary outcome variables were collected in a figure for comparison of studies in relation to the third review aim.

## 3. Results

### 3.1. Databases, Search Structure and Study Selection

**Figure 2:**
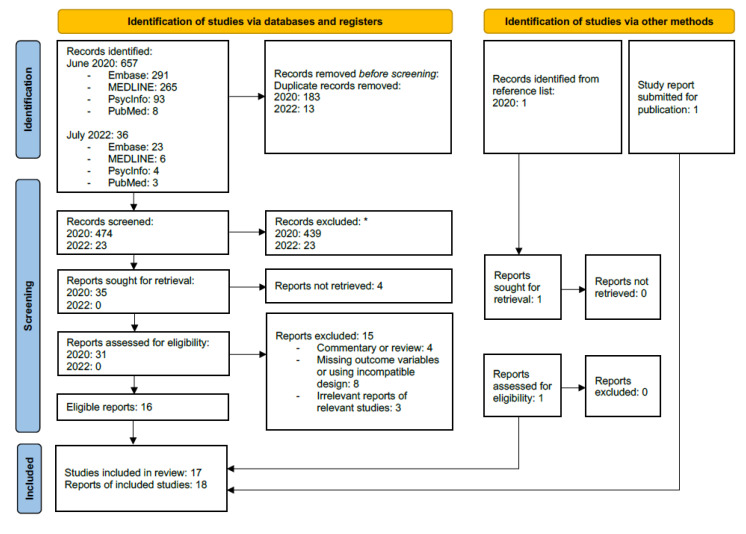
PRISMA 2020 flow diagram for new systematic reviews, adapted from Page et al. (2020) [40] under the terms of the Creative Commons Attribution License. * No automation tools were used.

The first systematic search identified 657 records (Figure 2). Of these, 182 were duplicates, and a total of 474 records were screened. After exclusion of 439 records based on the information available in title and abstract, full-text reports were sought for 35 records. Four of these could not be retrieved [44,45,46,47]. Fifteen more were excluded: four articles were letters, commentaries or reviews [48,49,50,51]; eight publications were reports of original studies, but did not have post treatment measures, did not use ECT, used treatment that involved Trp administration or Trp depletion, or did not measure Trp or kynurenines [52,53,54,55,56,57,58,59]; three articles were reports of eligible studies (same as D’Elia 1977b and Olajossy 2017) but did not provide additional information relevant to this review [60,61,62].

The second systematic search from July 2022 provided 36 records (Figure 2). Thirteen duplicates were removed, including two records that were present in the original search [37,56]. The remaining 23 records were screened and excluded based on title and abstract. Thus, no studies or reports were included from this search.

Outside of the systematic searches, one record was found from scanning reference lists from other included reports and was assessed for eligibility [63]. Additionally, a study report in submission was also eligible for inclusion [64].

### 3.2. Included Studies

**Figure 3:**
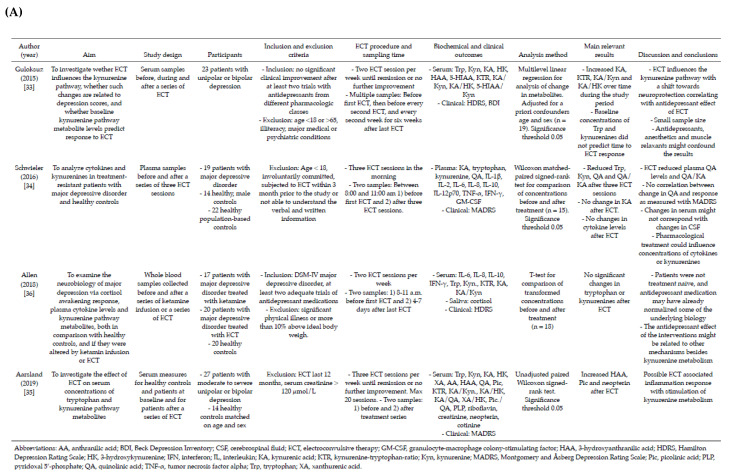

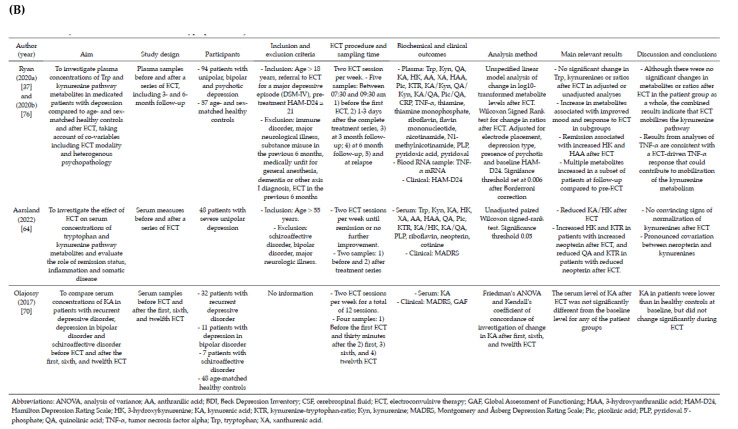

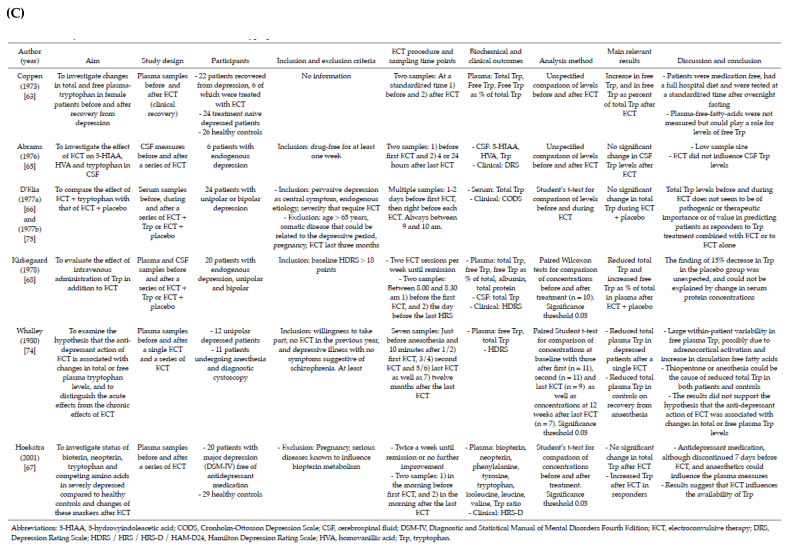

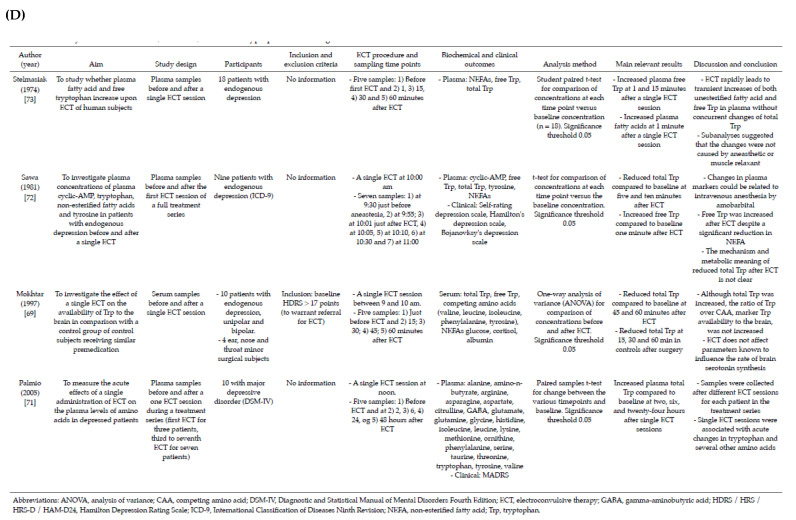
Summary of included studies: Studies of tryptophan and kynurenines after a series of ECT. (**A**,**B**) Summary of included studies: Studies of tryptophan and kynurenines after a series of ECT. (**C**) Summary of included studies : Studies of tryptophan after a series of ECT. (**D**) Summary of in-cluded studies : Studies of tryptophan after a single ECT.

Nineteen reports of seventeen studies were included [33,34,35,36,37,63,64,65,66,67,68,69,70,71,72,73,74,75,76]. Previously unpublished follow-up measures for 12 controls in Aarsland 2019 were also included. Together, these studies contained repeated measures or analyses of change in Trp or kynurenines for 386 patients with depression treated with ECT and 27 controls. The studies are presented in detail in Figure 3, and Supplementary Table S1 shows all collected biomarker concentrations (primary outcome variables).

### 3.3. Baseline Concentrations and Changes in Levels of Tryptophan, Kynurenines and Ratios after ECT

**Figure 4:**
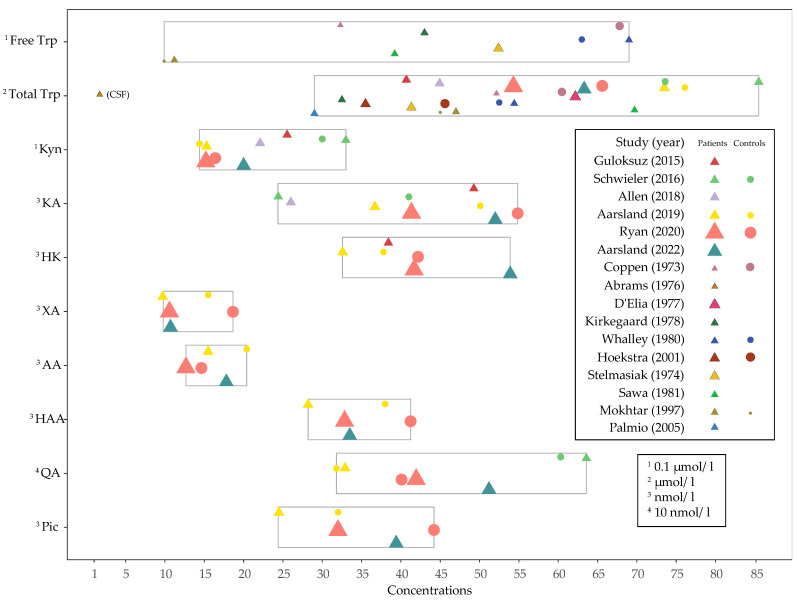
Baseline concentrations of Trp and kynurenines in patients and controls for each study. The size of each symbol represents the number of measured samples. The vertical position of the symbols within each biomarkers box corresponds with the order of the studies in the legend. See Supplementary Table S1 for detailed information on all collected outcome concentrations. References: Guloksuz (2015) [33], Schwieler (2016) [34], Allen (2018) [36], Aarsland (2019) [35], Ryan (2020) [37], Aarsland (2022) [64], Coppen (1973) [63], Abrams (1976) [65], D’Elia (1977) [66], Kirkegaard (1978) [68], Whalley (1980) [74], Hoekstra (2001) [67], Stelmasiak (1974) [73], Sawa (1981) [72], Mokhtar (1997) [69], Palmio (2005) [71].

Baseline concentrations of Trp and kynurenines for patients and controls are presented in Figure 4.

**Figure 5:**
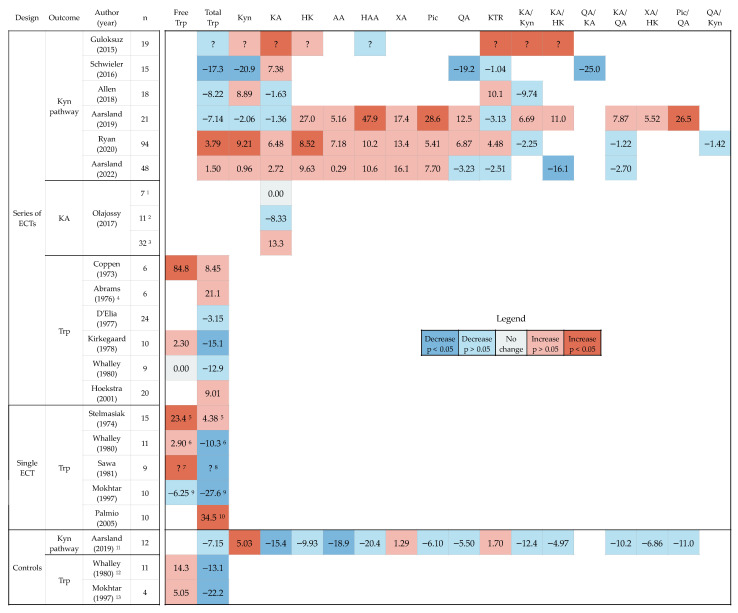
Percentage change in tryptophan, kynurenines and ratios after ECT. Percentage change was calculated based on concentrations before and after ECT collected from the included studies, except for Ryan (2020) [37] and Aarsland (2022) [64], where changes in percentage were collected from the reports. See Supplementary Table S1 for a detailed overview of the collected outcomes from each of the reviewed studies. The question marks indicate that the size of change was unknown due to missing data on concentrations. Comments on participant diagnosis, sample type, and sample timing: 1 schizoaffective disorder, 2 depression in bipolar disorder, 3 recurrent depressive disorder; 4 cerebrospinal fluid samples; samples collected 5 15 min after ECT, 6 10 min after first ECT, 7 1 min after ECT, 8 5 min after ECT, 9 60 min after ECT, 10 2 h after ECT, 11 with eight weeks between, 12 after recovery from anesthesia, and 13 15 min after start of surgery. Abbreviations: AA, anthranilic acid; ECT, electroconvulsive therapy; HAA, 3-hydroxyanthranilic acid; HK, 3-hydroxykynurenine; KA, kynurenic acid; KTR, kynurenine-tryptophan-ratio; Kyn, kynurenine; Pic, picolinic acid; QA, quinolinic acid; Trp, tryptophan; XA, xanthurenic acid. References: Guloksuz (2015) [33], Schwieler (2016) [34], Allen (2018) [36], Aarsland (2019) [35], Ryan (2020) [37], Aarsland (2022) [64], Olajossy (2017) [70], Coppen (1973) [63], Abrams (1976) [65], D’Elia (1977) [66], Kirkegaard (1978) [68], Whalley (1980) [74], Hoekstra (2001) [67], Stelmasiak (1974) [73], Sawa (1981) [72], Mokhtar (1997) [69], Palmio (2005) [71].

Percentage change after ECT of Trp, kynurenines and kynurenine pathway ratios are shown in Figure 5. Free Trp, total Trp, Kyn, KA and KTR were available in an adequate number of studies to utilize vote counting based on effect direction (exact binomial test, binom.test).

Free Trp was measured before and after ECT in seven studies. Setting statistical significance aside, five out of seven studies reported increased concentrations of free Trp after ECT. Vote counting based on these effect directions did not reject the null hypothesis that increase and decrease in free Trp after ECT were equally likely (71.4% cases of increase (95% confidence interval (95%CI): 29.1% to 96.3%), *p* = 0.45). For total Trp, seven studies found increased concentration while ten studies found decreased concentration after ECT, and as with free Trp, the null hypothesis was not rejected (35.3% cases of increase (95%CI: 18.4% to 67.1%), *p* = 0.41). Seven studies investigated concentrations of one or more kynurenines before and after a series of ECT. Kyn was measured in six of these studies, four of which found increased concentrations after ECT (66.7% cases of increase (95%CI: 22.3 to 95.7), *p* = 0.69). KA was the only metabolite measured in all seven studies, one of which investigated change in three patient groups separately. It was increased in five out of nine analyses (55.6% cases of increase (95%CI: 21.2% to 86.3%), *p* = 1). Finally, KTR increased after ECT in three studies and decreased in three others (50% cases of increase (95%CI: 11.8% to 88.2%), *p* = 1.00). Thus, there was no overall evidence of an effect of ECT on levels of Trp, Kyn, KA or KTR.

The three studies that included the largest panel of kynurenines all found trends of increase in HK, AA, HAA, XA and Pic. A fourth study also found increased HK and reduced HAA after ECT. However, neither of these kynurenines, nor QA or any of the pathway ratios, were reported in a sufficient number of studies to perform a vote counting.

Three studies had repeated measures from controls (Figure 5). In Whalley et al., eleven patients undergoing cystoscopy served as controls and had significant reduction in total Trp after anesthesia [74]. In Mokhtar et al., four anesthesia controls had significant reduction in total Trp 15 min after start of surgery [69]. In Aarsland et al., healthy controls (n = 12), with no intervention, had significant reduction in Kyn, KA and AA in follow-up samples collected eight weeks after baseline [35].

### 3.4. Factors That Can Affect Analyses of Tryptophan and Kynurenines

**Figure 6:**
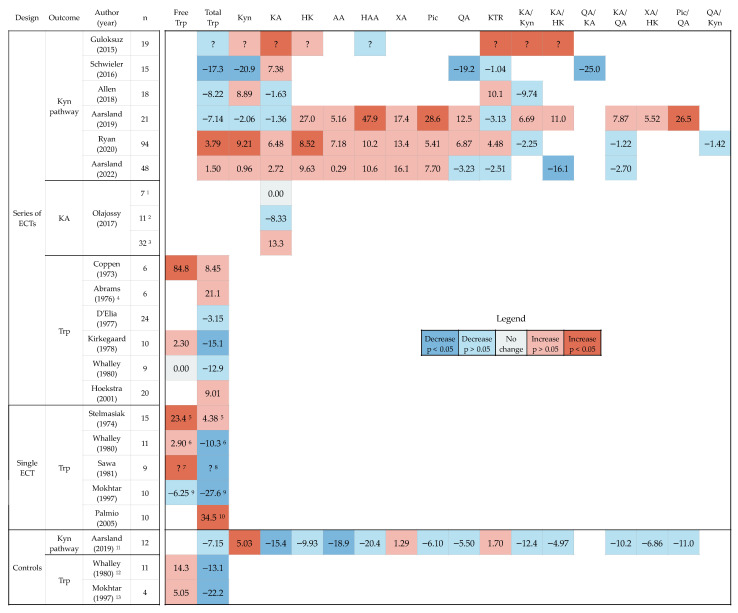
Simplified overview of the review studies’ declaration and handling of factors that can affect analyses of tryptophan and kynurenines in the context of ECT. The table is limited to factors that have been included in at least one of the reviewed studies. See Supplementary Table S2 for a detailed version with more information about the reported data for each study. Abbreviations: BMI, body mass index; CAA, competing amino acid; ECT, electroconvulsive therapy; NEFA, non-esterified fatty acid. References: Guloksuz (2015) [33], Schwieler (2016) [34], Allen (2018) [36], Aarsland (2019) [35], Ryan (2020) [37], Aarsland (2022) [64], Olajossy (2017) [70], Coppen (1973) [63], Abrams (1976) [65], D’Elia (1977) [66], Kirkegaard (1978) [68], Whalley (1980) [74], Hoekstra (2001) [67], Stelmasiak (1974) [73], Sawa (1981) [72], Mokhtar (1997) [69], Palmio (2005) [71].

Figure 6 (simplified version, see Section 4.2.2) and Supplementary Table S2 (detailed version) show a summary of factors known to be associated with either Trp-kynurenine metabolism, ECT response, or both and that were extracted from the included studies. The tables were limited to include factors reported in at least one of the reviewed studies. Declaration and investigations of these factors as possible mediators or confounders differed widely, with most studies only considering a few.

## 4. Discussion

### 4.1. Effect of ECT on Tryptophan, Kynurenines and Ratios

In this systematic review, the primary aim was to summarize changes in Trp and kynurenines and their ratios after ECT for patients with depression. We identified 17 studies that were eligible for inclusion. Sixteen of these reported measures of total Trp, seven reported measures of KA, four reported measures of QA, and three studies reported measures of a large panel of kynurenines and ratios. Vote counting based on direction of effect found no evidence for an effect of ECT on the levels of free Trp, total Trp, Kyn, KA or KTR. Three studies with a large panel of kynurenines all found trends of increase in HK, AA, HAA, XA and Pic, but these were too few for synthesis.

As described in the introduction, Trp availability, which is tightly associated with kynurenine pathway activation, and the balance between neuroactive kynurenines are two main aspects of Trp-kynurenine metabolism in relation to depression, and both were addressed by the reviewed studies. Pathway activation and balance have been suggested to be related, as inflammation induced activation of the pathway potentially causes a larger increase in HK and QA relative to the side-branch metabolite KA [2,77,78].

Considering Trp availability, our synthesis did not provide evidence of an effect of ECT on free Trp or total Trp. Measures of free Trp have been important for investigations of the role of Trp availability for cerebral serotonin production in depression. Increase in free Trp suggests that more Trp is available for metabolism [39], potentially facilitating more serotonin production that could contribute to depression symptom relief. Dependent on the cause of the increase in free Trp, various concomitant changes could be expected in total Trp. If due to strong Trp displacement from albumin, increased utilization of Trp could be reflected as a reduction in total Trp concentration [39]. Conversely, if the increase in free Trp was due to inhibition of Trp metabolizing enzymes, total Trp would also be expected to increase [39]. In the current review, three out of five studies with numerically increased free Trp after ECT also found numerically reduced total Trp after ECT. However, only one of three studies with significant increase in free Trp after ECT found significantly reduced total Trp. None of these studies on free Trp included kynurenines, so kynurenine pathway activation as a possible explanation for decreased Trp was not elucidated there. Instead, these studies investigated the possible role of other factors that could affect the balance of free and total Trp, including free fatty acids [69,72,73], competing amino acids [69,71], albumin concentration [68] and anesthesia [72,73,74], though without any conclusive results.

Kynurenine pathway activation was assessed in the six studies that included both Trp and kynurenines. As with free and total Trp, the results were inconsistent. The only study that found a significant decrease in total Trp after ECT also found a significant decrease in Kyn and QA, with a stable KTR [34], suggestive of an overall reduction in levels of Trp and kynurenines after ECT. In contrast, the single study that found significant increases in Trp after ECT also found significant increase in Kyn and HK, with a stable KTR [37], indicating a general increase in the availability of Trp. KTR was significantly altered after ECT in only one study (Guloksuz 2015). Here, it was increased, indicating increased pathway activity, with corresponding but non-significant increase in Kyn and decrease in Trp [33]. The most conspicuous pattern of change, however, was the trend of increased levels of HK, AA, HAA, XA and Pic in the three studies investigating a large panel of kynurenines, including the study with the most participants (94 patients with depression and 57 controls) [37]. Here, Trp was increased in two studies [37,64] and reduced in the third [35], and it is unclear if the apparent pathway activation was a consequence of increased Trp availability or, on the contrary, a reason for Trp decrease. As will be discussed further below, this aspect of analyses of change in kynurenine pathway activation could be elucidated through measures of factors affecting pathway enzymes, most importantly inflammatory markers, but potentially also glucocorticoids and vitamin B6 status. Ryan and colleagues found a significant decrease in TNF-α after ECT, suggesting reduced inflammation after ECT [37]. This is often associated with reduced kynurenine pathway activity and, therefore, seemingly inconsistent with the general trend of increase in pathway metabolites in this study. In the study of Schwieler and colleagues, several cytokines were measured, but none of them were changed significantly after ECT [34]. In two other studies, changes in the inflammatory marker neopterin coincided with changes in kynurenines, suggesting a role of altered cellular Th1-immune activation after ECT [35,64].

Markers related to the balance between neuroactive effects of kynurenines were available in seven studies. With a stable concentration of KA, and a significant reduction in QA and QA/KA, Schwieler 2016 pointed to a possible increased neuroprotection after ECT. Similarly, Guloksuz found increased KA, KA/Kyn and KA/QA after ECT, together with increased KTR. In contrast, two other studies found signs of lowered neuroprotection, with increased HK (and QA in adjusted analyses) in one [37] and reduced KA/HK in the other [64]. The three remaining studies found no significant changes related to pathway balance. From the synthesis, there was no evidence of an effect of ECT on KA. HK and QA, two main neuroactive metabolites suspected to cause neurotoxicity in relation to depression, were not available in a sufficient number of studies to perform a synthesis. Like with Trp, Kyn and KTR, KA and QA changed in both directions and in various degrees, as did various ratios used for estimating the balance between neuroactive effects: KA/Kyn, KA/HK and QA/KA (KA/QA).

The overall lack of consistent results coincides with findings in related biomarker literature, both in studies investigating the mechanisms of ECT (for a general overview of biomarkers for ECT, see [79,80]) and in studies analyzing the effect of other anti-depressant interventions on Trp-kynurenine metabolism (reviewed in [5]). The effect of ECT has been investigated on many other biochemical systems, some of which are tightly linked to kynurenine metabolism. Most importantly, change in the concentration of inflammatory markers after ECT has been the topic of many recent studies (reviewed in [81]). There, the overall results pointed to a short-term increase in inflammation markers IL-1 and IL-6 after ECT and a reduction in TNF-α and IL-6 levels in the long term. Similarly, there were findings of short-term increase in plasma cortisol after ECT, indicating an acute stress response, but also a long-term decrease in cortisol after a full treatment series. The review authors noted, however, that the studies were too few to be conclusive. Due to the activating role of cortisol, and the mutual regulation between kynurenines and inflammation systems, these fields are highly important for the investigations of kynurenine metabolism in relation to ECT. More and larger studies are therefore needed that investigate the relationship between ECT, inflammation and stress responses, including temporal aspects.

The effect of other anti-depressant treatments, including ketamine and selective serotonin reuptake inhibitors (SSRIs), on kynurenine metabolism has also been investigated. Like KA, ketamine is an antagonist of NMDAr and involved in regulation of immune activity [82]. The effects of ketamine on tryptophan metabolites have been investigated in a handful of studies (reviewed in [82]). One study showed increased levels of Kyn, KA and KA/Kyn and reduced levels of IDO and QA/Kyn after a series of ketamine infusions [83]. Another study, that was also included in the current review, compared the effects of ECT and ketamine [36] and found no effect of ketamine on kynurenines when looking at the whole ketamine treatment group. They found a trend, however, towards a decrease in Kyn at 2 h after the first infusion in ketamine responders. Similarly, a third study found increased KA and KA/Kyn in ketamine responders (Zhou 2018). The effects of ketamine on inflammatory markers have also been investigated, with some studies demonstrating decrease in peripheral levels of IL-1β, IL-6 and TNF-α [82]. A few studies provided data on kynurenines in relation to treatment with SSRIs (reviewed in [5]). Halaris and colleagues found reduced HK, QA and KA/QA in 15 patients with depression after 12 weeks of escitalopram treatment [84]. In a metabolomics study, Zhu and colleagues found reduced Kyn/melatonin and HK/melatonin in sertraline responders [85]. Finally, Mackay and colleagues found increased Trp at 6 and 12 weeks of fluoxetine therapy, but no change in kynurenines [86]. The same was found for a group of patients receiving counselling [86]. Like for ketamine, there were indications that SSRI treatment is associated with reduction in levels of inflammatory cytokines, specifically IL-1β and IL-6 [87].

Overall, there is still little solid evidence both of effect of ECT on other biochemical markers and of other anti-depressant treatments on kynurenine pathway metabolism. This general lack of convincing findings, both in relation to the mechanisms of ECT and the role of Trp metabolism in treatment of psychiatric disorders, is important as a context for interpreting the results of the current review. Most importantly, the lack of solid reproduced findings shows that the field is still in an exploratory phase, and that larger studies are probably needed to detect changes in kynurenines in relation to ECT. Moreover, the underlying mechanisms are complex and better understanding of the physiology, including normal variation, key determinants and other influential factors, are needed to unravel the role of Trp and the kynurenine pathway in this context.

### 4.2. Effect Modifiers and Mediators

It is apparent that clinical and methodological differences play a role when comparing studies and looking for overarching patterns. Due to large differences in patient characteristics and methods, comparing studies and summarizing findings is challenging. Given the supposed relationship between Trp and kynurenine pathway abnormalities and depression symptom severity, results of studies can vary, not only due to variables affecting Trp metabolism or measures of Trp metabolites, but potentially also due to differences in treatment response. As reported above, we collected information on some central factors (summarized in Figure 6/Supplementary Table S2) that can affect baseline concentrations, metabolite changes after ECT as well as the patients’ response to ECT. In the following, we discuss their relevance for Trp-kynurenine metabolism, and the implications for cross sectional comparisons and analyses of changes after ECT.

#### 4.2.1. The Kynurenine Pathway of Tryptophan Metabolism (Figure 1)

Trp is an essential amino acid, i.e., not synthesized in the human body, and is supplied from diet and protein degradation. In blood, about 90 percent of Trp is bound to albumin, and the remaining unbound fraction, free Trp, is available for metabolization [88]. Displacement of Trp from albumin increases the free fraction of Trp in blood, potentially increasing Trp availability for serotonin synthesis in the brain [89]. Trp levels in the central nervous system, however, are dependent on transport across the blood brain barrier (BBB) by L-type amino acid transporter (LAT1) [88]. Total Trp in serum is, therefore, dependent on nutritional supply, the concentration of albumin, the rate of binding to and release from albumin, transport into other tissues and its subsequent metabolization.

The first step of the kynurenine pathway is the conversion of Trp to formyl-kynurenine by one of two enzymes, tryptophan 2,3-dioxygenase (TDO) and indolamine 2,3-dioxygenase 1 (IDO1). TDO is activated by Trp itself and induced by glucocorticoids such as cortisol [89]. IDO1, on the other hand, is induced by pro-inflammatory cytokines, especially interferon gamma (IFN-γ) [90]. Formyl-kynurenine is rapidly converted to Kyn, which can be metabolized to HK by kynurenine monooxygenase (KMO), to KA by kynurenine aminotransferases (KATs) or to AA by kynureninase (KYNU). KYNU is also necessary for the further conversion of HK to 3-hydroxyanthralinic acid (HAA), which can be metabolized to QA or to picolinic acid (Pic). HK can also be converted to xanthurenic acid (XA) by KAT. Two B vitamins are central cofactors in the pathway. Pyridoxal 5′-phosphate (PLP), an active form of vitamin B 6, is cofactor of KAT and KYNU, and therefore necessary for the enzymatic steps leading to KA, AA, XA and HAA. Flavine adenine dinucleotide (FAD), the active form of vitamin B2, is cofactor of KMO and necessary for the conversion of Kyn to HK [91]. The activity of the pathway enzymes affects both upstream and downstream metabolites, as high activity consumes precursors, while low activity can cause precursor accumulation.

While most of Trp metabolism is handled by TDO in the liver, the activity of IDO1 in other tissues can increase dramatically under pro-inflammatory conditions [8]. Like IDO1, KMO is induced by IFN-γ [8]. Inflammation related induction of the pathway is often reflected in a higher KTR and can result in increased levels of HK and QA [2]. Kynurenine pathway metabolites are eliminated from the body mainly by renal excretion [92]. The concentration of kynurenines in the central nervous system is dependent on local metabolism and transport across the blood brain barrier (BBB). Like Trp, Kyn is transported across the BBB by LAT1. The concentration of IDO and TDO is substantially lower in the brain than in other tissues, and most of the local metabolism seems to be based on Kyn [24]. The ability of other kynurenines to cross the BBB is debated, but the primary view is that QA, and especially KA, cross poorly and that their concentration therefore is dependent on local metabolism in glial cells [24]. Like in other tissues, local inflammation in the brain can cause dramatic increase in IDO activity accompanied by increased production of kynurenine metabolites, both by glial cells and by infiltrating macrophages [8].

There are many factors that determine the metabolism of Trp through the kynurenine pathway and the fate of kynurenine metabolites and, hence, may affect the findings. Figure 6 (Supplementary Table S2 for detailed version) lists some of the most important factors that can affect Trp and the kynurenine pathway metabolism that have been measured, declared, or discussed in at least one of the reviewed studies. In the following, we will present and discuss these factors.

#### 4.2.2. Factors That Can Affect Levels of Tryptophan and Kynurenines

Inflammation

Reduced levels of Trp and increased levels of kynurenines have been demonstrated in a range of clinical conditions involving immune activation, including infection, autoimmune disorders, cancer, neurodegenerative diseases and more (reviewed in [93,94]). Neopterin is an inflammatory marker that, like the kynurenines, is increased in concentration upon IFN-γ stimulation, and it often correlates with KTR under inflammatory conditions [95,96,97,98]. Measures of neopterin are therefore useful to clarify whether observed changes in KTR, or kynurenines in general, are related to inflammation.

The influence of inflammation on kynurenine pathway activity is important in the context of ECT, as depression has consistently been shown to be associated with chronic low-grade inflammation (review and meta-analysis: [18], recent original paper on CRP: [20]). Increased concentrations of kynurenines in depressed patients as compared with controls may be confined to patients with elevated levels of inflammatory markers, such as CRP and TNF-α [99]. Moreover, the risk of depression is increased in patients undergoing cytokine treatment, and this association has been linked to activation of the kynurenine pathway [100]. Remission from depressive episodes has been shown to be accompanied by reduced levels of inflammation [101]. Investigations in patients with depression and in animal models of depression also suggest that antidepressant medications have anti-inflammatory effects [102,103]. Furthermore, as discussed above, a meta-analysis suggests that ECT may affect inflammation, with short-term increase in inflammatory markers after a single ECT, and a long-term decrease after a full treatment series [81]. Given the strong relationship between depression, inflammation and kynurenine metabolism, it must be suspected that changes in levels of kynurenines after ECT depends on altered inflammatory status. Inflammation markers should therefore be assessed when investigating change in kynurenines.

Among the original reports included in this review, five reported levels of inflammatory markers, including CRP, TNF-α, IL-6, IL-8, IL-10, IFN-γ and neopterin [34,35,36,37,64]. Four studies [33,36,37,67] explicitly stated the presence of infection, immune disorders or inflammatory diseases as an exclusion criterion, presumably to reduce some of the noise potentially introduced by this factor on analyses of kynurenines.

Age

In general, higher age is associated with lower levels of Trp (reviewed in [89]). In the largest study to date, investigating two distinct community-based age groups (age 45–46 years (n = 3723) and age 70–72 years (n = 3329)), higher age was also associated with higher levels of Kyn, AA, KA, HK and neopterin, as well as higher KTR [38]. Increased inflammation with higher age could be part of the explanation for this association [104]. CSF levels of neopterin and kynurenine pathway metabolites have been found to be positively correlated with each other and with age in 49 healthy women [105].

The mean or median age of patients included in the reviewed studies ranged from 40 to 73 years, indicating that the age differences could have contributed to the variability across studies.

Kidney Function

Several studies have shown high levels of kynurenines in individuals with reduced kidney function compared to individuals with normal kidney function [38,106,107,108,109]. In Theofylaktopoulou and colleagues’ work, levels above 95% of normal distribution for Kyn, AA, KA, HK, KTR and neopterin were all associated with kidney dysfunction. Positive correlations of KA and QA with creatinine have also been shown [107], consistent with the importance of renal excretion for elimination of these metabolites. However, kidney dysfunction may also be associated with increased immune activation with increased neopterin [110], CRP levels [107] and increased IDO activity [111]. Altered concentrations of kynurenines in the context of kidney dysfunction is therefore probably due to a combination of reduced excretion, increased activities of TDO [106,112], increased inflammation [107] and possibly other mechanisms. It has further been established that kidney disease is a risk factor for depression [113], and kidney disease incidence was associated with depression symptom scores in a recent prospective cohort study [114].

Of the included papers in this review, only two included measures of kidney function [35,64].

Body Mass Index (BMI)

There is an intricate relationship between metabolic regulation and the kynurenine pathway (reviewed in [115,116]). Trp, Kyn, KA, HK, HAA, XA and KTR have all been found to be higher in obese compared to normal-weight individuals [38]. Positive associations between BMI and KTR [117,118] and between BMI and Kyn [118] have also been documented. Furthermore, neopterin was also associated with BMI in 426 clinically defined healthy individuals [119], and IDO gene expression was found to be enhanced in adipose tissue of people with obesity [120]. A recent study found higher BMI in patients with MDD compared with healthy controls, though without association to QA or QA/KA, and the authors suggested that altered kynurenine metabolism in depression could be related to metabolic disturbances [121].

Two of the reviewed studies included data on BMI [36,37].

Sex

In the community-based study of Theofylaktopoulou et al., levels of Trp, Kyn, KA, HAA and XA were higher in men than in women [38]. In another cohort study of 2436 healthy young adults, Trp, Kyn, KA, AA and HAA were also higher in men [122]. There are also indications of differential responses of interventions on kynurenine metabolism in women and men, with women exhibiting greater changes in concentrations after Trp administration or IFN-treatment [25].

In general, the reviewed studies included more females than men, resulting in a stronger representation of females overall (242 vs 146).

B Vitamins, Tobacco and Alcohol

Low levels of PLP are associated with high concentrations of HK, and low concentrations of KA, AA, HAA and XA [123]. PLP concentration has been found to be reduced in many inflammatory conditions (reviewed in [124]), in smokers [125] and in subjects with high alcohol consumption [122]. Conversely, vitamin B6 supplements have been associated with lower HK concentrations [122]. High levels of nicotinamide (vitamin B3) can also inhibit TDO in a negative feedback mechanism [89]. There is an inverse association between smoking and concentration of several kynurenines [38] as well as KTR [126]. This could be related both to an anti-inflammatory effect of smoking and a reduction in circulating B-vitamin levels due to oxidative stress [125].

B-vitamin concentrations were reported in three studies [35,64,76], smoking in four studies [35,36,37,64] and alcohol consumption in one study [64].

Other Factors

Glucocorticoids, especially cortisol, are important inducers of TDO, and stress has long been recognized as a potential link between depression and kynurenine metabolism [15]. Both short-term increase and long-term decrease in cortisol have been described after ECT [81], potentially influencing peripheral metabolism through TDO activation. A number of other factors are also suggested to affect the availability of Trp for serotonin production, either through regulating the free fraction of Trp in blood, the transport across BBB or cell membranes or metabolization by TDO (reviewed in [89]). Trp can be displaced from albumin by non-esterified fatty acids (NEFAs), which could be increased in response to ECT as part of an acute stress response [89]. Some studies have also suggested that impaired liver function with reduced albumin production can cause increased circulating free Trp levels [89]. Dietary supply is essential for Trp levels, and intake of Trp has, for instance, been reported to be reduced in elderly with mild-to-moderate depression compared to healthy elderly controls [127]. Protein intake can, however, alter the ratio between Trp and other large neutral amino acids that compete for LAT1 transport, so called competing amino acids (CAAs). For example, administration of leucine, a CAA with high affinity for LAT1, has been shown to prevent depression-like behavior upon lipopolysaccharide stimulation in mice by blocking Kyn transport across the BBB [128]. Glucose has also been suggested to affect Trp levels through an inhibitory effect on TDO [89]. A range of common medications are furthermore suspected to affect levels of Trp and kynurenines, including anti-inflammatory drugs [129], oral contraceptives [130], salicylate [131], antirheumatic drugs [132] and more [39]. Still, knowledge about the clinical significance in humans of Trp displacement, TDO inhibition, various medications and the competitive action of CAAs for LAT1 transport is limited.

#### 4.2.3. Patient Characteristics

Patient characteristics are related both to expected treatment response and to the impact of the factors discussed above. Patients with depression constitute a highly heterogenous group and vary greatly between studies.

One main division is between major depressive disorder and depression in bipolar disorder, two closely related conditions that nevertheless have important differences, related to clinical characteristics, treatment methods, outcome and probably also etiology [133]. Furthermore, although alterations in kynurenine pathway metabolites have been found in patients with depression in general, many studies have suggested that pathway abnormalities may be more pronounced or relevant for various clinical subgroups. This includes depressed with high baseline concentrations of inflammatory markers [99,134], suicidal ideation [135,136,137,138] (reviewed in [138]) and psychotic features [139]. Similarly, comorbidity is also of importance, as kynurenine metabolism is often altered in somatic diseases (review in [94]), such as cancer, kidney disease, inflammatory disease, neurologic disease, diabetes [140], and psychiatric conditions such as schizophrenia [141]. Other relevant clinical characteristics, including depression severity [142], duration of depression [23], melancholy and anhedonia [143,144], cognitive function [145,146], and somatization [147], have all been related to inflammation and/or unbalance in kynurenine metabolism. Moreover, depression characteristics, age and inflammation are important predictors of treatment effect [148]. To address these aspects, stratification based on clinical data or correlation between kynurenines and clinical or biochemical scores were commonly applied in sub-analyses in the reviewed studies, though with limited statistical power.

Finally, medication is another important aspect of the study population. Whether patients are medication naïve or on anti-depressant medication could affect the response to ECT [149] and possibly kynurenine metabolism itself, for instance, through TDO inhibition [150]. This topic was central to the methodology and discussion of several of the reviewed studies. In general, most patients received treatment with antidepressants, antipsychotics or mood stabilizers during the study period, with limited statistical strength to draw any conclusions about the role of medication.

#### 4.2.4. Intervention and Study Design

As with patient characteristics, variation in study design and treatment delivery could contribute to differences in results. The method of ECT delivery varies between studies and could affect response [151] and potentially contribute to the variation in observed changes in Trp and its metabolites. The number of ECT sessions is a crucial factor since it is the main difference between two types of study design, single versus treatment series, and since the length of a series typically varies from patient to patient. It could be a factor acting directly onto the biomarker levels, as well as a proxy for treatment effect. Patients with a delayed treatment response of ECT are likely to receive more sessions. This could potentially lead to larger effects on inflammation and kynurenine metabolism in non-responders.

The medication used in context of ECT, anesthesia and muscle relaxants, could conceivably affect the Trp and kynurenine levels, and this was a topic discussed in several of the reviewed reports. Whalley and colleagues discussed these medications as the potential cause of change in Trp in both patients and controls [74]. Stelmasiak and Curzon concluded, based on the results of sub-analyses, that anesthetics did not play a significant role for changes in free or total Trp [73]. Less commonly discussed was the potential role of fasting, which could be substantial if blood samples are collected with different fasting length, such as in the single ECT design. Given the dietary dependency of Trp and the effect of cortisol on TDO, both Trp and kynurenines could conceivably show diurnal and seasonal variation. Collecting blood in the morning or in the evening, or at different seasons, could potentially yield different concentrations. There could also be an effect of meals received at the clinic or improved nutritional status as a consequence of reduced symptom burden or other treatment (e.g., hospitalization). Moreover, time between last ECT and post-treatment blood sampling is possibly of high importance when analyzing kynurenines after ECT. In the current review, the post-treatment sample time varied substantially both within and between studies that declared this information. Systematic sampling time differences could be an explanation both for baseline differences between patients and controls, and for changes over time, both in patients receiving therapy and for healthy controls. This is relevant, not only for investigations into the short-term effect that ECT by itself exerts on Trp metabolism, but also for analyses of changes related to reduced symptom severity. For instance, follow-up samples could reveal important biomarker alterations, such as the significant changes in a sub-group of patients at 3 months follow-up after ECT in the work of Ryan and colleagues [37].

Treatment response is another important measure in this type of study. Given the hypothesis of association between symptom severity and levels of kynurenines, changes after ECT are expected to be more pronounced in responders or remitters than in non-responders or non-remitters. Stratification by treatment effect, or analyses of correlations between changes in biochemical and clinical measures, could reveal differences in kynurenine changes after ECT that would otherwise be concealed. These strategies were utilized in several of the reviewed studies [34,35,36,64,67].

#### 4.2.5. Summary of the Role of Factors That Can Influence Analyses of Tryptophan and Kynurenines in the Context of ECT

This review indicates that many factors that are important for analyses of Trp-kynurenine metabolism are often not standardized, measured or reported in studies that investigate changes in these metabolites after ECT. The impact of these factors may vary according to study design. They can have an especially large impact when comparing groups of participants, such as baseline concentrations in patients and healthy controls. Controls are often selected to match patients on age and sex, but inflammation, kidney function, nutritional status, BMI, somatic disease and use of medication can potentially be important effect modifiers. For example, the observed differences in KA between 1100 depressed patients and 642 healthy controls were no longer significant after adjustment for age, sex, education, smoking status, alcohol consumption and chronic diseases [99].

In studies that utilize repeated measures, determinants of Trp and kynurenines should have limited effect on the analyses of change if they are stable throughout the treatment series. However, some important factors, such as inflammation, stress, B vitamin levels and medication, can change during the study period and could affect the outcome measures. Moreover, such changes could be an effect of ECT and, therefore, not readily adjusted for in statistical analyses without introducing bias. Additionally, it is still not clear to what degree the baseline concentrations of Trp or kynurenines affect the potential for change or treatment response. Many of the variables discussed above are potential determinants of baseline concentrations. As part of the focus on Trp availability as essential for cerebral serotonin production, two of the review studies investigated whether administration of the essential amino acid could be beneficial to the treatment effect of ECT [66,68]. While the use of Trp administration in depression treatment is controversial [116], the underlying question of the importance of baseline concentrations for remission remains unanswered. Some of the reviewed studies investigated the relationship between baseline levels of Trp/kynurenines and variables such as treatment response and pre- and post-treatment inflammatory marker levels and symptom scores [33,34,37,66]. Such analyses are important to shed light on the clinical role of baseline levels, for instance, as predictors of clinical response, but should also take into account the determinants of kynurenines to avoid confounded results.

The heterogeneity of depression and differences between studies in terms of diagnoses and clinical characteristics of included patients are well-known challenges in the search for biomarkers. Diversity in patient characteristics could contribute to differences between studies in baseline measures and changes after treatment, due to both variable weight of the factors discussed above and differences in treatment outcome. Consequently, the generalizability of each study’s findings could be limited. Especially for the purpose of evaluating the comparability of studies in the context reviews and meta-analyses, it is important that information is available for the most relevant and influential factors.

### 4.3. Challenges Regarding the Clinical Use of Tryptophan and Kynurenine Measures

Some of the variables discussed above may reduce the precision of change estimates or even cause misleading results. However, even with sufficient handling of such factors, there are additional challenges regarding the interpretation and application of Trp and kynurenine measures in a clinical setting. These are, especially: (1) insufficient knowledge on normal ranges and variability, (2) uncertainty regarding the value of blood measures as opposed to CSF measures in the context of neuropsychiatric disorders, and (3) difficulties relating to study design and statistical analyses, including a lack of methods to interpret changes in the pathway as an interactive network instead of single markers.

#### 4.3.1. Normal Ranges and Variability

Community studies shows that the normal range of Trp and kynurenine pathway metabolite concentrations are quite wide, with Trp ranging from 41.6 to 98.2 μmol/L, Kyn from 0.94 to 2.86 μmol/L and KA from 20.4–93.2 nmol/L [38,152]. Extreme values are related specially to kidney function, BMI and smoking [38]. Looking at the baseline levels of the studies included in the current review (Figure 3), there was also large variation in baseline concentrations of Trp and kynurenines, both for patients and controls.

Clinically harmful ranges of kynurenines are not well established, and it is not known what a given concentration of kynurenines means for an individual’s health. Abnormal levels are usually defined in each individual study, based on comparisons between patients and groups of healthy individuals. However, given the wide normal range and the large spread of concentration means in the reviewed studies, it seems unreliable, at least for small studies, to use control groups as a reference point for determining whether biomarker concentrations in patients are abnormal and in what direction they may change. Instead, population studies and meta-analyses should be used to provide points of reference.

Similarly, there are important challenges related to interpretation of change. In the current review, three included studies found significant changes in controls groups: in two studies samples were collected before and after anesthesia [69,74], and in the third, at baseline and at eight-week follow-up without any intervention [35]. These control groups were small (n = 4, 11 and 12 respectively), but the results suggest that the study design has important weaknesses. While the first two hint to a role of anesthesia and fasting in this type of study design, the changes in the third study were unexpected and the reasons unclear.

Investigations of metabolite levels depend on these concentrations being relatively stable over time, so that any observed changes can be attributed to the intervention rather than normal individual variation. Levels of Trp and kynurenines have been investigated with plasma measures in two cohorts without intervention, one with two samples 1–2 years apart (n = 40), and another with two samples 3.5 years apart (n = 402–545) [152]. Here, intraclass correlation coefficients (ICCs) were used to evaluate how much of the total sample variance was attributable to within-person variance as opposed to between-person variance. A high ICC indicates that the concentration of metabolite is quite stable if measured at two or more time points from the same individual, and the total sample variance is mainly due to concentration differences between individuals. High ICCs are preferable in intervention studies, as large within-person variance makes it difficult to distinguish the intervention effect from the normal individual variations. In the samples taken 3.5 years apart, Kyn, HK, KA, XA, AA and HAA all changed significantly. With ICCs corresponding to a reproducibility of fair-to-good (0.4–0.75), this study indicates that a substantial portion of the total variation was due to within-person variance. To our knowledge, there are no published studies on changes over a shorter period of time, mimicking clinical therapy trials. However, shorter time between samples and larger number of sample sizes both contribute to higher ICC and more reliable estimation of the intervention effect.

Alternatively, control groups that follow the same study structure as the depressed patients, only without the intervention, could provide a reference for normal variation over the timespan of the study [33,69]. This could also be useful for correction for the effect of factors relating to treatment, such as fasting and anesthesia. Such control groups could, for instance, be patients referred to procedures involving anesthesia.

#### 4.3.2. Peripheral and Central Concentrations

Among the studies reviewed here, only two collected CSF samples (Abrams (1976), Kirkegaard (1978)). The problem with using peripheral blood versus cerebrospinal fluid measures and the question of the relevance of blood samples are commonly discussed in studies of biomarkers in relation to neuropsychiatric disorders, including kynurenine metabolism. There is a general lack of studies investigating kynurenines in CSF. More and larger CSF studies could be important to study the biology of psychiatric disorders. However, recent studies point to a high correlation between serum and CSF levels, in healthy individuals, but also in depression [121,153,154] and other conditions such as Alzheimer’s disease [155].

#### 4.3.3. Research Questions, Study Design and Methods of Analysis

There were two main categories of studies included in this review: studies that investigated changes after a single ECT and those that investigated changes after a series of ECT. These two designs present two quite different approaches to the topic of Trp metabolism in relation to ECT. The former considers the effect of ECT independent of the anti-depressant effect, while the latter addresses the changes in depression symptoms that follows from the treatment.

For investigating the first mechanism, one or a few ECT sessions with blood measures before and after each session might suffice to investigate changes in concentrations related to the effect of ECT. Here, it is important that patients are matched, and that each patient receive the same numbers of ECT sessions, preferably with the equivalent settings, so that the study exposure is as similar as possible between subjects. Although the results are intrinsically linked to the diagnostic criteria for referral to ECT, the patients’ clinical response is not the focus. This design is especially vulnerable, however, to intervention related effects such as anesthesia and fasting. To shed light on the second mechanism, ECT serves as a convenient setting that can bring about dramatic changes in depression symptom levels. Here, the clinical response is an essential variable and should preferably be closely monitored. However, observed covariation, or change in response groups, is not easily separated from the direct effect of ECT considered using the first approach. Therefore, the two designs should preferably be combined and the two mechanisms addressed together in the same study, with samples collected both before and after single sessions and complete treatment series (such as in [74]).

Analytical methods have evolved during the period covered in this review. While the earlier studies mainly utilized fluorescence-based detection, recent studies have mainly used liquid chromatography combined with mass spectrometry (Supplementary Table S3). Differences in analytical procedures may have contributed to differences in results. However, such effects are likely of minor relevance compared to variations in sample collection and handling and in patient and control populations.

Finally, all the included studies in this review considered Trp or kynurenine pathway changes through single metabolites or ratios of two metabolites. Given the large number of metabolites in the pathway and their mutual dependence, a systems biology approach may be warranted as a complement to investigations of each metabolite in isolation.

### 4.4. Suggestions for Future Studies

The relationship between depression, ECT and the kynurenine metabolism is complex, and as put forward in this review, the current study groups are too small to reliably detect the effect of ECT on Trp and kynurenines. Future studies should investigate changes in Trp and kynurenines while reporting, considering and adjusting for factors that could affect Trp metabolism, ECT outcome or both. Studies should seek to include larger patient groups with standardized intervention, fasting and timepoints for sampling and matched control groups that follow the same procedures and timeline. In such analyses, kynurenines should be considered interdependent, and attempts should be made to analyze changes at the pathway level, not only in single metabolites or ratios. Besides the studies on ECT, studies are also needed that investigate between- and within-subject variability in kynurenines under normal physiological conditions, as well as the effect of fasting. Future studies should also analyze changes in light of response or remission status and consider if there are clinical subgroups in which alterations in Trp metabolism could be a more decisive aspect than in others, e.g., older patients. Additionally, a focus on dimensional scores as opposed to diagnoses could reduce variation between study populations and aid the search for generalizable results regarding the effects of ECT. To ensure comparability between studies, inclusion and exclusion criteria should be clearly described, and studies should strive for open datasets for transparency and to enable meta-analyses.

### 4.5. Strengths and Limitations

This review was based on a systematic literature search in four databases using a wide selection of relevant terms, with a Supplementary search in June 2022. No exclusion criteria were used in the search process. In addition, references of included studies were scanned for studies not found in the systematic search. Still, since the search only assessed title and abstract, studies may have been overlooked. In the synthesis of this review, changes in biomarkers were presented using percentage change. Alternative ways of analyzing changes could have yielded more or other types of information. Effect sizes could have been calculated based on mean and SD, and the difference between timepoints could have been evaluated in relation to the standard error to estimate clinical significance of the reported changes. Furthermore, more attention could have been given to analyses in subgroups of patients, for example, response or remission subgroups, or to correlation analyses of change in biomarkers in relation to change in symptom scores. Finally, the vote counting synthesis method does not consider size or significance of change, nor the quality of the included studies [42]. However, conclusions drawn from this material, even based on more advanced synthesis methods, would have high risk of bias given the large heterogeneity between studies, small study groups and the exploratory nature of this field.

## 5. Conclusions

In this systematic review, there was no overall evidence of change in Trp, kynurenines or ratios after ECT. This could reflect that the kynurenine pathway is not altered by this intervention. Alternatively, it may be due to limitations of the cited studies, such as the relatively low number of participants in each study, and the challenge of isolating the effect of ECT from the influence of other factors such as inflammation, stress and medication. Additionally, patients with depression are a heterogenous group, and differences in pathophysiology, clinical characteristics, treatment response and baseline concentrations of biomarkers could be of great importance. Finally, there is limited knowledge about the between- and within-individual variability in kynurenine metabolism and about the effect of fasting and the significance of differences in blood sample timing. However, despite the challenges involved, it is important to continue investigating the role of kynurenine metabolism in depression treatment, as this pathway could be crucial for understanding the pathophysiology of mood disorders and contains several potentially important targets for therapeutic interventions.

## Data Availability

Not applicable.

## Supplementary Materials

The Table following supporting information can be downloaded at https://www.mdpi.com/article/10.3390/ph15111439/s1: Table S1: Reported levels of tryptophan, kynurenines, ratios and related biomarkers before and after a series of ECT and corresponding analyses of change; Table S2: Detailed overview of the reviewed studies’ declaration and handling of factors that can affect analyses of tryptophan and kynurenines in the context of ECT; Table S3: Data on storage and analytical procedures in the reviewed studies. Document S1: Syntax for systematic literature search, july 2022.

## Abbreviations

AA	anthranilic acid
BMI	body mass index
CAA	competing amino acid
ECT	electroconvulsive therapy
FAD	flavin adenine dinucleotide
HAA	3-hydroxyanthranilic acid
HK	3-hydroxykynurenine
ICC	intraclass correlation coefficient
IDO	indoleamine 2,3-dioxygenase
IFN	interferon; KA, kynurenic acid
KYNU	kynureninase
Kyn	kynurenine
KAT	kynurenine aminotransferase
KMO	kynurenine monooxygenase
KTR	kynurenine-tryptophan-ratio
NEFA	non-esterified fatty acid
NMDAr	N-methyl-D-aspartate receptor
Pic	picolinic acid
PLP	pyridoxal 5‘-phosphate
QA	quinolinic acid
SSRI	selective serotonin reuptake inhibitor
TDO	tryptophan 2,3-dioxygenase
TNF	tumor necrosis factor
Trp	tryptophan
XA	xanthurenic acid

## References

[1] Arnone D., Saraykar S., Salem H., Teixeira A.L., Dantzer R., Selvaraj S. (2018). Role of Kynurenine pathway and its metabolites in mood disorders: A systematic review and meta-analysis of clinical studies. Neurosci. Biobehav. Rev..

[2] Savitz J. (2020). The kynurenine pathway: A finger in every pie. Mol. Psychiatry.

[3] Wirthgen E., Hoeflich A., Rebl A., Gunther J. (2017). Kynurenic Acid: The Janus-Faced Role of an Immunomodulatory Tryptophan Metabolite and Its Link to Pathological Conditions. Front. Immunol..

[4] Ogyu K., Kubo K., Noda Y., Iwata Y., Tsugawa S., Omura Y., Wada M., Tarumi R., Plitman E., Moriguchi S. (2018). Kynurenine pathway in depression: A systematic review and meta-analysis. Neurosci. Biobehav. Rev..

[5] Reus G.Z., Jansen K., Titus S., Carvalho A.F., Gabbay V., Quevedo J. (2015). Kynurenine pathway dysfunction in the pathophysiology and treatment of depression: Evidences from animal and human studies. J. Psychiatr Res..

[6] Ogawa S., Fujii T., Koga N., Hori H., Teraishi T., Hattori K., Noda T., Higuchi T., Motohashi N., Kunugi H. (2014). Plasma L-tryptophan concentration in major depressive disorder: New data and meta-analysis. J. Clin. Psychiatry.

[7] Massudi H., Grant R., Guillemin G.J., Braidy N. (2012). NAD+ metabolism and oxidative stress: The golden nucleotide on a crown of thorns. Redox Rep..

[8] Moffett J.R., Arun P., Puthillathu N., Vengilote R., Ives J.A., Badawy A.A., Namboodiri A.M. (2020). Quinolinate as a Marker for Kynurenine Metabolite Formation and the Unresolved Question of NAD(+) Synthesis During Inflammation and Infection. Front. Immunol..

[9] Vecsei L., Szalardy L., Fulop F., Toldi J. (2013). Kynurenines in the CNS: Recent advances and new questions. Nat. Rev. Drug Discov..

[10] Tanaka M., Toth F., Polyak H., Szabo A., Mandi Y., Vecsei L. (2021). Immune Influencers in Action: Metabolites and Enzymes of the Tryptophan-Kynurenine Metabolic Pathway. Biomedicines.

[11] Mor A., Tankiewicz-Kwedlo A., Krupa A., Pawlak D. (2021). Role of Kynurenine Pathway in Oxidative Stress during Neurodegenerative Disorders. Cells.

[12] Cervenka I., Agudelo L.Z., Ruas J.L. (2017). Kynurenines: Tryptophan’s metabolites in exercise, inflammation, and mental health. Science.

[13] O’Farrell K., Harkin A. (2017). Stress-related regulation of the kynurenine pathway: Relevance to neuropsychiatric and degenerative disorders. Neuropharmacology.

[14] Platten M., Nollen E.A.A., Rohrig U.F., Fallarino F., Opitz C.A. (2019). Tryptophan metabolism as a common therapeutic target in cancer, neurodegeneration and beyond. Nat. Rev. Drug Discov..

[15] Oxenkrug G. (2013). Serotonin-kynurenine hypothesis of depression: Historical overview and recent developments. Curr. Drug Targets.

[16] Moir A.T., Eccleston D. (1968). The effects of precursor loading in the cerebral metabolism of 5-hydroxyindoles. J. Neurochem..

[17] Bender D.A. (1983). Effects of a dietary excess of leucine on the metabolism of tryptophan in the rat: A mechanism for the pellagragenic action of leucine. Br. J. Nutr..

[18] Kohler C.A., Freitas T.H., Maes M., de Andrade N.Q., Liu C.S., Fernandes B.S., Stubbs B., Solmi M., Veronese N., Herrmann N. (2017). Peripheral cytokine and chemokine alterations in depression: A meta-analysis of 82 studies. Acta Psychiatr. Scand..

[19] Smith K.J., Au B., Ollis L., Schmitz N. (2018). The association between C-reactive protein, Interleukin-6 and depression among older adults in the community: A systematic review and meta-analysis. Exp. Gerontol..

[20] Pitharouli M.C., Hagenaars S.P., Glanville K.P., Coleman J.R.I., Hotopf M., Lewis C.M., Pariante C.M. (2021). Elevated C-Reactive Protein in Patients With Depression, Independent of Genetic, Health, and Psychosocial Factors: Results From the UK Biobank. Am. J. Psychiatry.

[21] Martinez-Cengotitabengoa M., Carrascon L., O’Brien J.T., Diaz-Gutierrez M.J., Bermudez-Ampudia C., Sanada K., Arrasate M., Gonzalez-Pinto A. (2016). Peripheral Inflammatory Parameters in Late-Life Depression: A Systematic Review. Int. J. Mol. Sci..

[22] Lapin I.P. (1973). Kynurenines as probable participants of depression. Pharmakopsychiatr. Neuropsychopharmakol..

[23] Myint A.M., Kim Y.K. (2003). Cytokine-serotonin interaction through IDO: A neurodegeneration hypothesis of depression. Med. Hypotheses.

[24] Schwarcz R., Bruno J.P., Muchowski P.J., Wu H.Q. (2012). Kynurenines in the mammalian brain: When physiology meets pathology. Nat. Rev. Neurosci..

[25] Maes M., Leonard B.E., Myint A.M., Kubera M., Verkerk R. (2011). The new ‘5-HT’ hypothesis of depression: Cell-mediated immune activation induces indoleamine 2,3-dioxygenase, which leads to lower plasma tryptophan and an increased synthesis of detrimental tryptophan catabolites (TRYCATs), both of which contribute to the onset of depression. Prog. Neuropsychopharmacol. Biol. Psychiatry.

[26] Perkins M.N., Stone T.W. (1982). An iontophoretic investigation of the actions of convulsant kynurenines and their interaction with the endogenous excitant quinolinic acid. Brain Res..

[27] Carpenedo R., Pittaluga A., Cozzi A., Attucci S., Galli A., Raiteri M., Moroni F. (2001). Presynaptic kynurenate-sensitive receptors inhibit glutamate release. Eur. J. Neurosci..

[28] Guillemin G.J. (2012). Quinolinic acid, the inescapable neurotoxin. FEBS J..

[29] Okuda S., Nishiyama N., Saito H., Katsuki H. (1996). Hydrogen peroxide-mediated neuronal cell death induced by an endogenous neurotoxin, 3-hydroxykynurenine. Proc. Natl. Acad. Sci. USA.

[30] Bartoli F., Misiak B., Callovini T., Cavaleri D., Cioni R.M., Crocamo C., Savitz J.B., Carra G. (2021). The kynurenine pathway in bipolar disorder: A meta-analysis on the peripheral blood levels of tryptophan and related metabolites. Mol. Psychiatry.

[31] Marx W., McGuinness A.J., Rocks T., Ruusunen A., Cleminson J., Walker A.J., Gomes-da-Costa S., Lane M., Sanches M., Diaz A.P. (2021). The kynurenine pathway in major depressive disorder, bipolar disorder, and schizophrenia: A meta-analysis of 101 studies. Mol. Psychiatry.

[32] Kocki T., Wnuk S., Kloc R., Kocki J., Owe-Larsson B., Urbanska E.M. (2012). New insight into the antidepressants action: Modulation of kynurenine pathway by increasing the kynurenic acid/3-hydroxykynurenine ratio. J. Neural. Transm..

[33] Guloksuz S., Arts B., Walter S., Drukker M., Rodriguez L., Myint A.M., Schwarz M.J., Ponds R., van Os J., Kenis G. (2015). The impact of electroconvulsive therapy on the tryptophan-kynurenine metabolic pathway. Brain Behav. Immun..

[34] Schwieler L., Samuelsson M., Frye M.A., Bhat M., Schuppe-Koistinen I., Jungholm O., Johansson A.G., Landen M., Sellgren C.M., Erhardt S. (2016). Electroconvulsive therapy suppresses the neurotoxic branch of the kynurenine pathway in treatment-resistant depressed patients. J. Neuroinflammation.

[35] Aarsland T.I., Leskauskaite I., Midttun O., Ulvik A., Ueland P.M., Oltedal L., Erchinger V.J., Oedegaard K.J., Haavik J., Kessler U. (2019). The effect of electroconvulsive therapy (ECT) on serum tryptophan metabolites. Brain Stimul..

[36] Allen A.P., Naughton M., Dowling J., Walsh A., O’Shea R., Shorten G., Scott L., McLoughlin D.M., Cryan J.F., Clarke G. (2018). Kynurenine pathway metabolism and the neurobiology of treatment-resistant depression: Comparison of multiple ketamine infusions and electroconvulsive therapy. J. Psychiatr. Res..

[37] Ryan K.M., Allers K.A., McLoughlin D.M., Harkin A. (2020). Tryptophan metabolite concentrations in depressed patients before and after electroconvulsive therapy. Brain Behav. Immun..

[38] Theofylaktopoulou D., Midttun O., Ulvik A., Ueland P.M., Tell G.S., Vollset S.E., Nygard O., Eussen S.J. (2013). A community-based study on determinants of circulating markers of cellular immune activation and kynurenines: The Hordaland Health Study. Clin. Exp. Immunol..

[39] Badawy A.A. (2010). Plasma free tryptophan revisited: What you need to know and do before measuring it. J. Psychopharmacol..

[40] Page M.J., McKenzie J.E., Bossuyt P.M., Boutron I., Hoffmann T.C., Mulrow C.D., Shamseer L., Tetzlaff J.M., Akl E.A., Brennan S.E. (2021). The PRISMA 2020 statement: An updated guideline for reporting systematic reviews. BMJ.

[41] Campbell M., McKenzie J.E., Sowden A., Katikireddi S.V., Brennan S.E., Ellis S., Hartmann-Boyce J., Ryan R., Shepperd S., Thomas J. (2020). Synthesis without meta-analysis (SWiM) in systematic reviews: Reporting guideline. BMJ.

[42] McKenzie J.E., Brennan S.E. Chapter 12: Synthesizing and presenting findings using other methods. In: Higgins JPT, Thomas J, Chandler J, Cumpston M, Li T, Page MJ, Welch VA (editors). Cochrane Handbook for Systematic Reviews of Interventions version 6.3 (updated February 2022). Cochrane, 2022. www.training.cochrane.org/handbook.

[43] Team R.C. (2019). R: A Language and Environment for Statistical Computing. 2019. R Core Team (2019) R: A Language and Environment for Statistical Computing. R Foundation for Statistical Computing, Vienna, Austria. https://www.R-project.org/.

[44] Barkai A.I. (1985). Combined electroconvulsive and drug therapy. Compr. Ther..

[45] Gerne B. (1984). Treatment of depression. Sven. Farm. Tidskr..

[46] Heymans C., De Schaepdryver A.F., Delaunois A.L., Piette Y. (1964). Pharmacology of Electroshock. Med. Mon..

[47] Heymans C., Deschaepdryver A.F., Delaunois A.L., Piette Y. (1964). Drugs and Electroshock. Riforma Med..

[48] Broadhurst A.D. (1970). L-tryptophan verses E.C.T. Lancet.

[49] Cocheme M.A. (1970). L-tryptophan versus E.C.T. Lancet.

[50] Shaw D.M. (1970). L-tryptophan in depression. Lancet.

[51] Carroll B.J., Mowbray R.M., Davies B. (1970). L-tryptophan in depression. Lancet.

[52] Bech P., Kirkegaard C., Bock E., Johannesen M., Rafaelsen O.J. (1978). Hormones, electrolytes, and cerebrospinal fluid proteins in manic-melancholic patients. Neuropsychobiology.

[53] Cassidy F., Murry E., Weiner R.D., Carroll B.J. (1997). Lack of relapse with tryptophan depletion following successful treatment with ECT. Am. J. Psychiatry.

[54] Cassidy F., Weiner R.D., Cooper T.B., Carroll B.J. (2010). Combined catecholamine and indoleamine depletion following response to ECT. Br. J. Psychiatry.

[55] Herrington R.N., Bruce A., Johnstone E.C., Lader M.H. (1974). Comparative trial of L tryptophan and E.C.T. in severe depressive illness. Lancet.

[56] Kranaster L., Hoyer C., Mindt S., Neumaier M., Muller N., Zill P., Schwarz M.J., Moll N., Lutz B., Bindila L. (2020). The novel seizure quality index for the antidepressant outcome prediction in electroconvulsive therapy: Association with biomarkers in the cerebrospinal fluid. Eur. Arch. Psychiatry Clin. Neurosci..

[57] Carroll B.J., Mowbray R.M., Davies B. (1970). Sequential comparison of L-tryptophan with E.C.T. in severe depression. Lancet.

[58] Coppen A., Brooksbank B.W., Eccleston E., Peet M., White S.G. (1974). Tryptophan metabolism in depressive illness. Psychol. Med..

[59] Coppen A., Shaw D.M., Malleson A., Eccleston E., Gundy G. (1965). Tryptamine metabolism in depression. Br. J. Psychiatry.

[60] Olajossy M., Olajossy B., Potembska E., Skoczen N., Wnuk S., Urbanska E. (2018). Differences in the dynamics of changes in the concentration of kynurenic acid in the blood serum of depressed patients treated with electroconvulsive therapy. Psychiatr.

[61] D’Elia G., Lehmann J., Raotma H. (1978). Influence of tryptophan on memory functions in depressive patients treated with unilateral ECT. Acta Psychiatr. Scand..

[62] D’Elia G., Lehmann J., Raotma H. (1979). Bimodal distribution of serum tryptophan level. Acta Psychiatr. Scand..

[63] Coppen A., Eccleston E.G., Peet M. (1973). Total and free tryptophan concentration in the plasma of depressive patients. Lancet.

[64] Aarsland T.I.M., Haavik J., Ulvik A., Ueland P.M., Dols A., Kessler U. (2022). The effect of electroconvulsive therapy (ECT) on serum kynurenine pathway metabolites in late-life depression.

[65] Abrams R., Essman W.B., Taylor M.A., Fink M. (1976). Concentration of 5-hydroxyindoleacetic acid, homovanillic acid, and tryptophan in the cerebrospinal fluid of depressed patients before and after ECT. Biol. Psychiatry.

[66] D’Elia G., Lehmann J., Raotma H. (1977). Evaluation of the combination of tryptophan and ECT in the treatment of depression. II. Biochemical analysis. Acta Psychiatr. Scand..

[67] Hoekstra R., van den Broek W.W., Fekkes D., Bruijn J.A., Mulder P.G., Pepplinkhuizen L. (2001). Effect of electroconvulsive therapy on biopterin and large neutral amino acids in severe, medication-resistant depression. Psychiatry Res..

[68] Kirkegaard C., Moller S.E., Bjorum N. (1978). Addition of L-tryptophan to electroconvulsive treatment in endogenous depression. A double-blind study. Acta Psychiatr Scand..

[69] Mokhtar A.S., Morgan C.J., Bradley D.M., Badawy A.A.B. (1997). No early effects of electroconvulsive therapy on tryptophan metabolism and disposition in endogenous depression. Biol. Psychiatry.

[70] Olajossy M., Olajossy B., Wnuk S., Potembska E., Urbanska E. (2017). Blood serum concentrations of kynurenic acid in patients diagnosed with recurrent depressive disorder, depression in bipolar disorder, and schizoaffective disorder treated with electroconvulsive therapy. Psychiatr. Pol..

[71] Palmio J., Huuhka M., Saransaari P., Oja S.S., Peltola J., Leinonen E., Suhonen J., Keranen T. (2005). Changes in plasma amino acids after electroconvulsive therapy of depressed patients. Psychiatry Res..

[72] Sawa Y. (1981). The effect of electroconvulsive therapy on plasma cyclic-AMP, non-esterified fatty acid, tryptophan and tyrosine in depression. Keio J. Med..

[73] Stelmasiak Z., Curzon G. (1974). Effect of electroconvulsive therapy on plasma unesterified fatty acid and free tryptophan concentrations in man. J. Neurochem..

[74] Whalley L.J., Yates C.M., Christie J.E. (1980). Effect of electroconvulsive therapy (ECT) on plasma tryptophan. Psychol. Med..

[75] D’Elia G., Lehmann J., Raotma H. (1977). Evaluation of the combination of tryptophan and ECT in the treatment of depression. I. Clinical analysis. Acta Psychiatr. Scand..

[76] Ryan K.M., Allers K.A., Harkin A., McLoughlin D.M. (2020). Blood plasma B vitamins in depression and the therapeutic response to electroconvulsive therapy. Brain Behav. Immun. Health.

[77] Wichers M.C., Koek G.H., Robaeys G., Verkerk R., Scharpe S., Maes M. (2005). IDO and interferon-alpha-induced depressive symptoms: A shift in hypothesis from tryptophan depletion to neurotoxicity. Mol. Psychiatry.

[78] Campbell B.M., Charych E., Lee A.W., Moller T. (2014). Kynurenines in CNS disease: Regulation by inflammatory cytokines. Front. Neurosci..

[79] Pinna M., Manchia M., Oppo R., Scano F., Pillai G., Loche A.P., Salis P., Minnai G.P. (2018). Clinical and biological predictors of response to electroconvulsive therapy (ECT): A review. Neurosci. Lett..

[80] Maffioletti E., Carvalho Silva R., Bortolomasi M., Baune B.T., Gennarelli M., Minelli A. (2021). Molecular Biomarkers of Electroconvulsive Therapy Effects and Clinical Response: Understanding the Present to Shape the Future. Brain Sci..

[81] Yrondi A., Sporer M., Peran P., Schmitt L., Arbus C., Sauvaget A. (2018). Electroconvulsive therapy, depression, the immune system and inflammation: A systematic review. Brain Stimul..

[82] Kopra E., Mondelli V., Pariante C., Nikkheslat N. (2021). Ketamine’s effect on inflammation and kynurenine pathway in depression: A systematic review. J. Psychopharmacol..

[83] Kadriu B., Farmer C.A., Yuan P., Park L.T., Deng Z.D., Moaddel R., Henter I.D., Shovestul B., Ballard E.D., Kraus C. (2021). The kynurenine pathway and bipolar disorder: Intersection of the monoaminergic and glutamatergic systems and immune response. Mol. Psychiatry.

[84] Halaris A., Myint A.M., Savant V., Meresh E., Lim E., Guillemin G., Hoppensteadt D., Fareed J., Sinacore J. (2015). Does escitalopram reduce neurotoxicity in major depression?. J. Psychiatr. Res..

[85] Zhu H., Bogdanov M.B., Boyle S.H., Matson W., Sharma S., Matson S., Churchill E., Fiehn O., Rush J.A., Krishnan R.R. (2013). Pharmacometabolomics of response to sertraline and to placebo in major depressive disorder—Possible role for methoxyindole pathway. PLoS ONE.

[86] Mackay G.M., Forrest C.M., Christofides J., Bridel M.A., Mitchell S., Cowlard R., Stone T.W., Darlington L.G. (2009). Kynurenine metabolites and inflammation markers in depressed patients treated with fluoxetine or counselling. Clin. Exp. Pharmacol. Physiol..

[87] Hannestad J., DellaGioia N., Bloch M. (2011). The effect of antidepressant medication treatment on serum levels of inflammatory cytokines: A meta-analysis. Neuropsychopharmacology.

[88] Skorobogatov K., De Picker L., Verkerk R., Coppens V., Leboyer M., Muller N., Morrens M. (2021). Brain Versus Blood: A Systematic Review on the Concordance Between Peripheral and Central Kynurenine Pathway Measures in Psychiatric Disorders. Front. Immunol..

[89] Badawy A.A. (2017). Tryptophan availability for kynurenine pathway metabolism across the life span: Control mechanisms and focus on aging, exercise, diet and nutritional supplements. Neuropharmacology.

[90] Yoshida R., Imanishi J., Oku T., Kishida T., Hayaishi O. (1981). Induction of pulmonary indoleamine 2,3-dioxygenase by interferon. Proc. Natl. Acad. Sci. USA.

[91] Theofylaktopoulou D., Ulvik A., Midttun O., Ueland P.M., Vollset S.E., Nygard O., Hustad S., Tell G.S., Eussen S.J. (2014). Vitamins B2 and B6 as determinants of kynurenines and related markers of interferon-gamma-mediated immune activation in the community-based Hordaland Health Study. Br. J. Nutr..

[92] Badawy A.A., Guillemin G. (2019). The Plasma [Kynurenine]/[Tryptophan] Ratio and Indoleamine 2,3-Dioxygenase: Time for Appraisal. Int. J. Tryptophan. Res..

[93] Schrocksnadel K., Wirleitner B., Winkler C., Fuchs D. (2006). Monitoring tryptophan metabolism in chronic immune activation. Clin. Chim. Acta.

[94] Strasser B., Becker K., Fuchs D., Gostner J.M. (2017). Kynurenine pathway metabolism and immune activation: Peripheral measurements in psychiatric and co-morbid conditions. Neuropharmacology.

[95] Deac O.M., Mills J.L., Gardiner C.M., Shane B., Quinn L., Midttun O., McCann A., Meyer K., Ueland P.M., Fan R. (2016). Serum Immune System Biomarkers Neopterin and Interleukin-10 Are Strongly Related to Tryptophan Metabolism in Healthy Young Adults. J. Nutr..

[96] Fuchs D., Moller A.A., Reibnegger G., Werner E.R., Werner-Felmayer G., Dierich M.P., Wachter H. (1991). Increased endogenous interferon-gamma and neopterin correlate with increased degradation of tryptophan in human immunodeficiency virus type 1 infection. Immunol. Lett..

[97] Midttun O., Ulvik A., Ringdal Pedersen E., Ebbing M., Bleie O., Schartum-Hansen H., Nilsen R.M., Nygard O., Ueland P.M. (2011). Low plasma vitamin B-6 status affects metabolism through the kynurenine pathway in cardiovascular patients with systemic inflammation. J. Nutr..

[98] Sperner-Unterweger B., Neurauter G., Klieber M., Kurz K., Meraner V., Zeimet A., Fuchs D. (2011). Enhanced tryptophan degradation in patients with ovarian carcinoma correlates with several serum soluble immune activation markers. Immunobiology.

[99] Milaneschi Y., Allers K.A., Beekman A.T.F., Giltay E.J., Keller S., Schoevers R.A., Sussmuth S.D., Niessen H.G., Penninx B. (2021). The association between plasma tryptophan catabolites and depression: The role of symptom profiles and inflammation. Brain Behav. Immun..

[100] Hunt C., Macedo E.C.T., Suchting R., de Dios C., Cuellar Leal V.A., Soares J.C., Dantzer R., Teixeira A.L., Selvaraj S. (2020). Effect of immune activation on the kynurenine pathway and depression symptoms—A systematic review and meta-analysis. Neurosci. Biobehav. Rev..

[101] Dahl J., Ormstad H., Aass H.C., Malt U.F., Bendz L.T., Sandvik L., Brundin L., Andreassen O.A. (2014). The plasma levels of various cytokines are increased during ongoing depression and are reduced to normal levels after recovery. Psychoneuroendocrinology.

[102] Dionisie V., Filip G.A., Manea M.C., Manea M., Riga S. (2021). The anti-inflammatory role of SSRI and SNRI in the treatment of depression: A review of human and rodent research studies. Inflammopharmacology.

[103] Vojvodic J., Mihajlovic G., Vojvodic P., Radomirovic D., Vojvodic A., Vlaskovic-Jovicevic T., Peric-Hajzler Z., Matovic D., Dimitrijevic S., Sijan G. (2019). The Impact of Immunological Factors on Depression Treatment—Relation Between Antidepressants and Immunomodulation Agents. Open Access Maced. J. Med. Sci..

[104] Sorgdrager F.J.H., Naude P.J.W., Kema I.P., Nollen E.A., Deyn P.P. (2019). Tryptophan Metabolism in Inflammaging: From Biomarker to Therapeutic Target. Front. Immunol..

[105] de Bie J., Guest J., Guillemin G.J., Grant R. (2016). Central kynurenine pathway shift with age in women. J. Neurochem..

[106] Saito K., Fujigaki S., Heyes M.P., Shibata K., Takemura M., Fujii H., Wada H., Noma A., Seishima M. (2000). Mechanism of increases in L-kynurenine and quinolinic acid in renal insufficiency. Am. J. Physiol. Renal. Physiol.

[107] Schefold J.C., Zeden J.P., Fotopoulou C., von Haehling S., Pschowski R., Hasper D., Volk H.D., Schuett C., Reinke P. (2009). Increased indoleamine 2,3-dioxygenase (IDO) activity and elevated serum levels of tryptophan catabolites in patients with chronic kidney disease: A possible link between chronic inflammation and uraemic symptoms. Nephrol. Dial. Transplant..

[108] Debnath S., Velagapudi C., Redus L., Thameem F., Kasinath B., Hura C.E., Lorenzo C., Abboud H.E., O’Connor J.C. (2017). Tryptophan Metabolism in Patients With Chronic Kidney Disease Secondary to Type 2 Diabetes: Relationship to Inflammatory Markers. Int. J. Tryptophan. Res..

[109] Zhao J. (2013). Plasma kynurenic acid/tryptophan ratio: A sensitive and reliable biomarker for the assessment of renal function. Ren. Fail..

[110] Lhee H.Y., Kim H., Joo K.J., Jung S.S., Lee K.B. (2006). The clinical significance of serum and urinary neopterin levels in several renal diseases. J. Korean Med. Sci..

[111] Mor A., Kalaska B., Pawlak D. (2020). Kynurenine Pathway in Chronic Kidney Disease: What’s Old, What’s New, and What’s Next?. Int. J. Tryptophan. Res..

[112] Pawlak D., Tankiewicz A., Matys T., Buczko W. (2003). Peripheral distribution of kynurenine metabolites and activity of kynurenine pathway enzymes in renal failure. J. Physiol. Pharmacol..

[113] Palmer S., Vecchio M., Craig J.C., Tonelli M., Johnson D.W., Nicolucci A., Pellegrini F., Saglimbene V., Logroscino G., Fishbane S. (2013). Prevalence of depression in chronic kidney disease: Systematic review and meta-analysis of observational studies. Kidney Int..

[114] Liu M., Zhang Y., Yang S., Wu Q., Ye Z., Zhou C., He P., Zhang Y., Hou F.F., Qin X. (2022). Bidirectional relations between depression symptoms and chronic kidney disease. J. Affect. Disord..

[115] Dadvar S., Ferreira D.M.S., Cervenka I., Ruas J.L. (2018). The weight of nutrients: Kynurenine metabolites in obesity and exercise. J. Intern. Med..

[116] Le Floc’h N., Otten W., Merlot E. (2011). Tryptophan metabolism, from nutrition to potential therapeutic applications. Amino. Acids.

[117] Mangge H., Summers K.L., Meinitzer A., Zelzer S., Almer G., Prassl R., Schnedl W.J., Reininghaus E., Paulmichl K., Weghuber D. (2014). Obesity-related dysregulation of the tryptophan-kynurenine metabolism: Role of age and parameters of the metabolic syndrome. Obesity.

[118] Favennec M., Hennart B., Caiazzo R., Leloire A., Yengo L., Verbanck M., Arredouani A., Marre M., Pigeyre M., Bessede A. (2015). The kynurenine pathway is activated in human obesity and shifted toward kynurenine monooxygenase activation. Obesity.

[119] Spencer M.E., Jain A., Matteini A., Beamer B.A., Wang N.Y., Leng S.X., Punjabi N.M., Walston J.D., Fedarko N.S. (2010). Serum levels of the immune activation marker neopterin change with age and gender and are modified by race, BMI, and percentage of body fat. J. Gerontol. A Biol. Sci. Med. Sci..

[120] Wolowczuk I., Hennart B., Leloire A., Bessede A., Soichot M., Taront S., Caiazzo R., Raverdy V., Pigeyre M., Consortium A. (2012). Tryptophan metabolism activation by indoleamine 2,3-dioxygenase in adipose tissue of obese women: An attempt to maintain immune homeostasis and vascular tone. Am. J. Physiol. Regul. Integr. Comp. Physiol..

[121] Paul E.R., Schwieler L., Erhardt S., Boda S., Trepci A., Kampe R., Asratian A., Holm L., Yngve A., Dantzer R. (2022). Peripheral and central kynurenine pathway abnormalities in major depression. Brain Behav. Immun..

[122] Deac O.M., Mills J.L., Shane B., Midttun O., Ueland P.M., Brosnan J.T., Brosnan M.E., Laird E., Gibney E.R., Fan R. (2015). Tryptophan catabolism and vitamin B-6 status are affected by gender and lifestyle factors in healthy young adults. J. Nutr..

[123] Ulvik A., Theofylaktopoulou D., Midttun O., Nygard O., Eussen S.J., Ueland P.M. (2013). Substrate product ratios of enzymes in the kynurenine pathway measured in plasma as indicators of functional vitamin B-6 status. Am. J. Clin. Nutr..

[124] Ueland P.M., McCann A., Midttun O., Ulvik A. (2017). Inflammation, vitamin B6 and related pathways. Mol. Aspects Med..

[125] Ulvik A., Ebbing M., Hustad S., Midttun O., Nygard O., Vollset S.E., Bonaa K.H., Nordrehaug J.E., Nilsen D.W., Schirmer H. (2010). Long- and short-term effects of tobacco smoking on circulating concentrations of B vitamins. Clin. Chem..

[126] Pertovaara M., Heliovaara M., Raitala A., Oja S.S., Knekt P., Hurme M. (2006). The activity of the immunoregulatory enzyme indoleamine 2,3-dioxygenase is decreased in smokers. Clin. Exp. Immunol..

[127] Chojnacki C., Poplawski T., Chojnacki J., Fila M., Konrad P., Blasiak J. (2020). Tryptophan Intake and Metabolism in Older Adults with Mood Disorders. Nutrients.

[128] Walker A.K., Wing E.E., Banks W.A., Dantzer R. (2019). Leucine competes with kynurenine for blood-to-brain transport and prevents lipopolysaccharide-induced depression-like behavior in mice. Mol. Psychiatry.

[129] Regan T., Gill A.C., Clohisey S.M., Barnett M.W., Pariante C.M., Harrison N.A., Consortium M.R.C.I., Hume D.A., Bullmore E.T., Freeman T.C. (2018). Effects of anti-inflammatory drugs on the expression of tryptophan-metabolism genes by human macrophages. J. Leukoc. Biol..

[130] Meier T.B., Drevets W.C., Teague T.K., Wurfel B.E., Mueller S.C., Bodurka J., Dantzer R., Savitz J. (2018). Kynurenic acid is reduced in females and oral contraceptive users: Implications for depression. Brain Behav. Immun..

[131] Paoletti R., Sirtori C., Spano P.F. (1975). Clinical relevance of drugs affecting tryptophan transport. Annu. Rev. Pharmacol..

[132] McArthur J.N., Dawkins P.D., Smith M.J., Hamilton E.B. (1971). Mode of action of antirheumatic drugs. Br. Med. J..

[133] Harrison P.J., Geddes J.R., Tunbridge E.M. (2018). The Emerging Neurobiology of Bipolar Disorder. Trends Neurosci..

[134] Carlier A., Berkhof J.G., Rozing M., Bouckaert F., Sienaert P., Eikelenboom P., Veerhuis R., Vandenbulcke M., Berkhof J., Stek M.L. (2019). Inflammation and remission in older patients with depression treated with electroconvulsive therapy; findings from the MODECT study(). J. Affect. Disord..

[135] Janelidze S., Mattei D., Westrin A., Traskman-Bendz L., Brundin L. (2011). Cytokine levels in the blood may distinguish suicide attempters from depressed patients. Brain Behav. Immun..

[136] Brundin L., Sellgren C.M., Lim C.K., Grit J., Palsson E., Landen M., Samuelsson M., Lundgren K., Brundin P., Fuchs D. (2016). An enzyme in the kynurenine pathway that governs vulnerability to suicidal behavior by regulating excitotoxicity and neuroinflammation. Transl. Psychiatry.

[137] Messaoud A., Mensi R., Douki W., Neffati F., Najjar M.F., Gobbi G., Valtorta F., Gaha L., Comai S. (2019). Reduced peripheral availability of tryptophan and increased activation of the kynurenine pathway and cortisol correlate with major depression and suicide. World J. Biol. Psychiatry.

[138] Bryleva E.Y., Brundin L. (2017). Kynurenine pathway metabolites and suicidality. Neuropharmacology.

[139] Wurfel B.E., Drevets W.C., Bliss S.A., McMillin J.R., Suzuki H., Ford B.N., Morris H.M., Teague T.K., Dantzer R., Savitz J.B. (2017). Serum kynurenic acid is reduced in affective psychosis. Transl. Psychiatry.

[140] Oxenkrug G.F. (2015). Increased Plasma Levels of Xanthurenic and Kynurenic Acids in Type 2 Diabetes. Mol. Neurobiol..

[141] Erhardt S., Schwieler L., Imbeault S., Engberg G. (2017). The kynurenine pathway in schizophrenia and bipolar disorder. Neuropharmacology.

[142] Elovainio M., Hurme M., Jokela M., Pulkki-Raback L., Kivimaki M., Hintsanen M., Hintsa T., Lehtimaki T., Viikari J., Raitakari O.T. (2012). Indoleamine 2,3-dioxygenase activation and depressive symptoms: Results from the Young Finns Study. Psychosom. Med..

[143] Gabbay V., Ely B.A., Babb J., Liebes L. (2012). The possible role of the kynurenine pathway in anhedonia in adolescents. J. Neural. Transm..

[144] Gabbay V., Liebes L., Katz Y., Liu S., Mendoza S., Babb J.S., Klein R.G., Gonen O. (2010). The kynurenine pathway in adolescent depression: Preliminary findings from a proton MR spectroscopy study. Prog. Neuropsychopharmacol. Biol. Psychiatry.

[145] Solvang S.H., Nordrehaug J.E., Tell G.S., Nygard O., McCann A., Ueland P.M., Midttun O., Meyer K., Vedeler C.A., Aarsland D. (2019). The kynurenine pathway and cognitive performance in community-dwelling older adults. The Hordaland Health Study. Brain Behav. Immun..

[146] Hestad K.A., Engedal K., Whist J.E., Farup P.G. (2017). The Relationships among Tryptophan, Kynurenine, Indoleamine 2,3-Dioxygenase, Depression, and Neuropsychological Performance. Front. Psychol..

[147] Maes M., Rief W. (2012). Diagnostic classifications in depression and somatization should include biomarkers, such as disorders in the tryptophan catabolite (TRYCAT) pathway. Psychiatry Res..

[148] Stippl A., Kirkgoze F.N., Bajbouj M., Grimm S. (2020). Differential Effects of Electroconvulsive Therapy in the Treatment of Major Depressive Disorder. Neuropsychobiology.

[149] Pluijms E.M., Kamperman A.M., Hoogendijk W.J., Birkenhager T.K., van den Broek W.W. (2021). Influence of an adjuvant antidepressant on the efficacy of electroconvulsive therapy: A systematic review and meta-analysis. Aust. New Zealand J. Psychiatry.

[150] Badawy A.A., Evans M. (1981). Inhibition of rat liver tryptophan pyrrolase activity and elevation of brain tryptophan concentration by administration of antidepressants. Biochem. Pharmacol..

[151] van Diermen L., van den Ameele S., Kamperman A.M., Sabbe B.C.G., Vermeulen T., Schrijvers D., Birkenhager T.K. (2018). Prediction of electroconvulsive therapy response and remission in major depression: Meta-analysis. Br. J. Psychiatry.

[152] Midttun O., Townsend M.K., Nygard O., Tworoger S.S., Brennan P., Johansson M., Ueland P.M. (2014). Most blood biomarkers related to vitamin status, one-carbon metabolism, and the kynurenine pathway show adequate preanalytical stability and within-person reproducibility to allow assessment of exposure or nutritional status in healthy women and cardiovascular patients. J. Nutr..

[153] Haroon E., Welle J.R., Woolwine B.J., Goldsmith D.R., Baer W., Patel T., Felger J.C., Miller A.H. (2020). Associations among peripheral and central kynurenine pathway metabolites and inflammation in depression. Neuropsychopharmacology.

[154] Sellgren C.M., Gracias J., Jungholm O., Perlis R.H., Engberg G., Schwieler L., Landen M., Erhardt S. (2019). Peripheral and central levels of kynurenic acid in bipolar disorder subjects and healthy controls. Transl. Psychiatry.

[155] Jacobs K.R., Lim C.K., Blennow K., Zetterberg H., Chatterjee P., Martins R.N., Brew B.J., Guillemin G.J., Lovejoy D.B. (2019). Correlation between plasma and CSF concentrations of kynurenine pathway metabolites in Alzheimer’s disease and relationship to amyloid-beta and tau. Neurobiol. Aging.

