# Supplementary material for: Changes in Tryptophan-Kynurenine Metabolism in Patients with Depression Undergoing ECT—A Systematic Review
Source: Pharmaceuticals (Basel). 2022 Nov 19;15(11):1439. doi: 10.3390/ph15111439 (PMC9694349; doi:10.3390/ph15111439)
Supplement: Supplementary file 1 [file pharmaceuticals-15-01439-s001.zip › Document S1.pdf]

Supplementary Document: Syntax for systematic literature search, july 2022

The syntax for the original literature search for june 2020 was identical to the one presented below. The number of records identified by each index term or free text in the syntax is indicated at the end of each line. Unlike in the original search, the results showen here were filtered on publication date.

Ovid MEDLINE(R) and Epub Ahead of Print, In-Process, In-Data-Review & Other Non-Indexed Citations and Daily <1946 to July 19, 2022>

1 (Kynurenine pathway or tryptophan kynurenine pathway or tryptophan kynurenine metabolism or tryptophan catabolism or tryptophan catabolite pathway or TRYCAT).mp. 2682
2 tryptophan.mp. 65112
3 kynurenine.mp. 6883
4 (kynurenine tryptophan ratio or KTR).mp. 817
5 (kynurenic acid or kynurenate or KYNA).mp. 4130
6 anthranilic acid.mp. 1776
7 (hydroxykynurenine or 3-hydroxykynurenine).mp. 831
8 xanthurenic acid.mp. 674
9 (hydroxyanthranilic acid or 3-hydroxyanthranilic acid).mp. 710
10 (quinolinic acid or QUIN).mp. 4048
11 picolinic acid.mp. 1106
12 tryptophan 2,3-dioxygenase.mp. 649
13 indoleamine 2,3-dioxygenase.mp. 4297
14 (kynureninase or kynurenine hydrolase or KYNU).mp. 407
15 kynurenine aminotransferase.mp. 311
16 kynurenine 3-monooxygenase.mp. 343
17 1 or 2 or 3 or 4 or 5 or 6 or 7 or 8 or 9 or 10 or 11 or 12 or 13 or 14 or 15 or 16 77159
18 exp tryptophan/ 35459
19 exp kynurenine/ 3876
20 exp kynurenic acid/ 2501
21 exp ortho-aminobenzoates/ 8070
22 exp xanthurenates/ 3030
23 exp 3-hydroxyanthranilic acid/ 340
24 exp quinolinic acid/ 1790
25 exp picolinic acids/ 3590
26 exp tryptophan oxygenase/ 1993
27 exp indoleamine-pyrrole 2,3,-dioxygenase/ 3261
28 exp kynurenine 3-monooxygenase/ 252
29 18 or 19 or 20 or 21 or 22 or 23 or 24 or 25 or 26 or 27 or 28 54079
30 17 or 29 89654
31 (Electroconvulsive or Electroshock or Electroconvulsive Therapy or Electroconvulsive Therapies or Electroshock Therapy or Electroshock Therapies or Electric Convulsive Therapy or Electric Convulsive Therapies or Electric Shock Therapy or Electric shock Therapies or Electroconvulsive Shock Therapy or Electroconvulsive Shock Therapies or Electroconvulsive shock or ECT).mp. 33799
32 exp Electroconvulsive Therapy/ 13965
33 31 or 32 33799
34 30 and 33 267
35 limit 34 to yr="2020 -Current" 6

Embase 1974 to 2022 July 19

1 (Kynurenine pathway or tryptophan kynurenine pathway or tryptophan kynurenine metabolism or tryptophan catabolism or tryptophan catabolite pathway or TRYCAT).mp. 3581
2 tryptophan.mp. 79946
3 kynurenine.mp. 9464
4 (kynurenine tryptophan ratio or KTR).mp. 1919
5 (kynurenic acid or kynurenate or KYNA).mp. 5093
6 anthranilic acid.mp. 4175
7 (hydroxykynurenine or 3-hydroxykynurenine).mp. 1152
8 xanthurenic acid.mp. 899
9 (hydroxyanthranilic acid or 3-hydroxyanthranilic acid).mp. 907
10 (quinolinic acid or QUIN).mp. 5581
11 picolinic acid.mp. 2210
12 tryptophan 2,3-dioxygenase.mp. 1813
13 indoleamine 2,3-dioxygenase.mp. 9057
14 (kynureninase or kynurenine hydrolase or KYNU).mp. 605
15 kynurenine aminotransferase.mp. 584
16 kynurenine 3-monooxygenase.mp. 553
17 1 or 2 or 3 or 4 or 5 or 6 or 7 or 8 or 9 or 10 or 11 or 12 or 13 or 14 or 15 or 16 100720
18 exp tryptophan/ 49648
19 exp kynurenine/ 7397
20 exp kynurenic acid/ 4162
21 exp anthranilic acid/ 1770
22 exp 3-hydroxykynurenine/ 905
23 exp xanthurenic acid/ 759
24 exp 3-hydroxyanthranilic acid/ 623
25 exp quinolinic acid/ 3530
26 exp picolinic acid/ 1189
27 exp tryptophan 2,3 dioxygenase/ 1647
28 exp indoleamine 2,3 dioxygenase/ 7720
29 exp kynureninase/ 451
30 exp Kynurenine 3 monooxygenase/ 520
31 exp Kynurenine aminotransferase/ 456
32 18 or 19 or 20 or 21 or 22 or 23 or 24 or 25 or 26 or 27 or 28 or 29 or 30 or 31 65535
33 17 or 32 100720
34 (Electroconvulsive or Electroshock or Electroconvulsive Therapy or Electroconvulsive Therapies or Electroshock Therapy or Electroshock Therapies or Electric Convulsive Therapy or Electric Convulsive Therapies or Electric Shock Therapy or Electric Shock Therapies or Electroconvulsive Shock Therapy or Electroconvulsive Shock Therapies or Electroconvulsive Shock or ECT).mp. 30370
35 exp Electroconvulsive Therapy/ 20071
36 34 or 35 30370
37 33 and 36 310
38 limit 37 to yr="2020 -Current" 23

APA PsycInfo 1806 to July Week 2 2022

1 (Kynurenine pathway or tryptophan kynurenine pathway or tryptophan kynurenine metabolism or tryptophan catabolism or tryptophan catabolite pathway or TRYCAT).mp. 491
2 tryptophan.mp. 4461
3 kynurenine.mp. 840
4 (kynurenine tryptophan ratio or KTR).mp. 47
5 (kynurenic acid or kynurenate or KYNA).mp. 747

6 anthranilic acid.mp. 35
7 (hydroxykynurenine or 3-hydroxykynurenine).mp. 98
8 xanthurenic acid.mp. 42
9 (hydroxyanthranilic acid or 3-hydroxyanthranilic acid).mp. 35
10 (quinolinic acid or QUIN).mp. 515
11 picolinic acid.mp. 28
12 tryptophan 2,3-dioxygenase.mp. 49
13 indoleamine 2,3-dioxygenase.mp. 252
14 (kynureninase or kynurenine hydrolase or KYNU).mp. 12
15 kynurenine aminotransferase.mp. 35
16 kynurenine 3-monooxygenase.mp. 54
17 1 or 2 or 3 or 4 or 5 or 6 or 7 or 8 or 9 or 10 or 11 or 12 or 13 or 14 or 15 or 16 5459
18 exp tryptophan/ 2384
19 17 or 18 5615
20 (Electroconvulsive or Electroshock or Electroconvulsive Therapy or Electroconvulsive Therapies or Electroshock Therapy or Electroshock Therapies or Electric Convulsive Therapy or Electric Convulsive Therapies or Electric Shock Therapy or Electric shock Therapies or Electroconvulsive Shock Therapy or Electroconvulsive Shock Therapies or Electroconvulsive shock or ECT).mp. 18654
21 exp Electroconvulsive Shock Therapy/ 7077
22 20 or 21 18654
23 19 and 22 101
24 limit 23 to yr="2020 -Current" 4

Pubmed from 2020/6/3 - 2022/7/20

((kynurenine pathway[Title/Abstract] OR tryptophan kynurenine pathway[Title/Abstract] OR tryptophan kynurenine metabolism[Title/Abstract] OR tryptophan catabolism[Title/Abstract] OR tryptophan catabolite pathway[Title/Abstract] OR TRYCAT[Title/Abstract] OR tryptophan[Title/Abstract] OR kynurenine[Title/Abstract] OR kynurenine tryptophan ratio[Title/Abstract] OR KTR[Title/Abstract] OR kynurenic acid[Title/Abstract] OR kynurenate[Title/Abstract] OR KYNA[Title/Abstract] OR anthranilic acid[Title/Abstract] OR hydroxyanthranilic acid[Title/Abstract] OR 3-hydroxyanthranilic acid[Title/Abstract] OR quinolinic acid[Title/Abstract] OR QUIN[Title/Abstract] OR picolinic acid[Title/Abstract] OR tryptophan 2,3,-dioxygenase[Title/Abstract] OR indoleamine 2,3-dioxygenase[Title/Abstract] OR kynureninase[Title/Abstract] OR kynurenine hydrolase[Title/Abstract] OR KYNU[Title/Abstract] OR kynurenine aminotransferase[Title/Abstract] OR kynurenine 3-monooxygenase) AND (electroconvulsive[Title/Abstract] OR electroshock[Title/Abstract] OR electroconvulsive therapy[Title/Abstract] OR electroconvulsive therapies[Title/Abstract] OR electroshock therapy[Title/Abstract] OR electroshock therapies[Title/Abstract] OR electric convulsive therapy[Title/Abstract] OR electric convulsive therapies[Title/Abstract] OR electric shock therapy[Title/Abstract] OR electric shock therapies[Title/Abstract] OR electroconvulsive shock therapy[Title/Abstract] OR electroconvulsive shock therapies[Title/Abstract] OR electroconvulsive shock[Title/Abstract] OR ECT[Title/Abstract]) 87

Filters applied: from 2020/6/4 - 2022/7/20 3
