# Supplementary material for: Changes in Tryptophan-Kynurenine Metabolism in Patients with Depression Undergoing ECT—A Systematic Review
Source: Pharmaceuticals (Basel). 2022 Nov 19;15(11):1439. doi: 10.3390/ph15111439 (PMC9694349; doi:10.3390/ph15111439)
Supplement: Supplementary file 1 [file pharmaceuticals-15-01439-s001.zip › Table S3.pdf]

Supplementary Table 3 Data on storage and analytical procedures in the reviewed studies

Author (year)	Storage	Analytical procedure	Articles cited
Guloksuz (2015)	"Serum was seperated and stored at -80ºC"	"High performance liquid chromatography (HPLC) was used []". Metabolites were detected "spectrophotometrically", "fluorimetrically" or "by UV detection".	Hervé et al. (1996) *Determination of tryptophan and its kynurenine pathway metabolites in human serum by high-performance liquid chromatography with simultaneous ultraviolet and fluorimetric detection.* J. Chromatogr. B 675, 157–161.<br><br>Oades et al. (2010a) *Attention-deficit hyperactivity disorder (ADHD) and glial integrity: S100B, cytokines and kynurenine metabolism-effects of medication.* Behav. Brain Funct. 6, 29.<br><br>Oades et al. (2010b) *Attention-deficit hyperactivity disorder (ADHD) and glial integrity: an exploration of associations of cytokines and kynurenine metabolites with symptoms and attention.* Behav. Brain Funct. 6, 32.
Schwieler (2016)	"Blood samples were centrifuged [], and plasma was collected and stored at −70 °C until analysis"	"Analysis of KYNA was measured by high-performance liquid chromatography (HPLC) with fluorescence detection."<br><br>"[For tryptophan, kynurenine and QUIN the] detection was performed using a [] mass spectrometer []."	Olsson et al. (2010) *Elevated levels of kynurenic acid in the cerebrospinal fluid of patients with bipolar disorder.* J Psychiatry Neurosci. 35 (3): 195–9.
Allen (2018)	"Samples were centrifuged immediately and frozen at −80 °C."	"[] analysis on the HPLC system (UV and FLD detection)."	Clarke et al. (2009) *Tryptophan degradation in irritable bowel syndrome: evidence of indoleamine 2,3-dioxygenase activation in a male cohort.* BMC Gastroenterol. 9 (1), 6.
Aarsland (2019)	"[] serum separated and stored at -80 °C until analysis."	"[] liquid chromatography-tandem mass spectrometry."	Midttun et al. (2009) *Quantitative profiling of biomarkers related to B-vitamin status, tryptophan metabolism and inflammation in human plasma by liquid chromatography/tandem mass spectrometry.* Rapid Commun Mass Spectrom 23 (9): 1371–1379.
Ryan (2020)	"Plasma was stored in aliquots at −80 °C until analysis."	"Liquid chromatography-tandem mass spectrometry"	Midttun et al. (2009) *Quantitative profiling of biomarkers related to B-vitamin status, tryptophan metabolism and inflammation in human plasma by liquid chromatography/tandem mass spectrometry.* Rapid Commun Mass Spectrom 23 (9): 1371–1379.<br><br>Midttun et al. (2013) *High-throughput, low-volume, multi-analyte quantification of plasma metabolites related to one-carbon metabolism using HPLC-MS/MS.* Anal. Bioanal. Chem. 405, 2009–2017.
Aarsland (2022)	Not described	"[] liquid chromatography / tandem mass spectrometry."	Midttun et al. (2009) *Quantitative profiling of biomarkers related to B-vitamin status, tryptophan metabolism and inflammation in human plasma by liquid chromatography/tandem mass spectrometry.* Rapid Commun Mass Spectrom 23 (9): 1371–1379.
Olajossy (2017)	"[] the supernatant was collected and frozen at −72°C."	"The content of KYNA in serum was assessed [] using a Varian Pro Star 210 liquid chromatograph []."	Turski et al (1988) *Identification and quantification of kynurenic acid in human brain tissue.* Brain Res. 454(1–2): 164–169.
Coppen (1973)	"[] samples of the ultra filtrate and plasma were deep-frozen."	"Free and total acid-soluble tryptophan were [] estimated by a modification of the method of Denckla and Dewey." (Spectrofluorometry)	Denckla and Dewey (1967) *The determination of tryptophan in plasma, liver and urine.* J. lab. clin. Med. 69, 160-169.
Abrams (1976)	"The first 8-10 ml of CSF was placed without preservative in a freezer at -20 C within 5 min of removal and was stored until analysis."	"A third aliquot was assayed for TRYP contend using an adaptation of a tissue assay for this aromativ amino acid (Tagliamonte et al., 1971)." (Spectrofluorometry)	Taglimonte et al. (1971) *Effect of psychotropic drugs and tryptophan in the rat brain.* J. Pharmacol. Exptl. Therap. 177: 475.
D'Elia (1977)	Not described	"Analyses of total L-TP concentrations were performed according to Denckla & Dewey (1967) []." (Spectrofluorometry)	Denckla and Dewey (1967) *The determination of tryptophan in plasma, liver and urine.* J. lab. clin. Med. 69, 160-169.

Kirkegaard (1978)	"The EDTA-2Na stabilized plasma was stored at -20ºC."	"[] the ultrafiltration and the determination of total and free tryptophan was carried out as described elsewhere (Møller et al. (1976))." (Spectrofluorometry)	Møller et al. (1976): Plasma amino acids as an index for subgroups in manic depressive psychosis: correlation to effect of tryptophan. Psychopharmacology 49, 205-213.<br><br>In which "The total acid-soluble and the free TRY were determined according to Denckla and Dewey (1967)"
Whalley (1980)	"Plasma and ultrafiltrate were stored at -20ºC until assayed for total and free tryptophan, respectively."	"Plasma and ultrafiltrate were stored at -20ºC until assayed for total and free tryptophan, respectively." (Spectrofluorometry)	Hess and Udenfreid (1959) *A fluorometric procedure for the estimatin of tryptamine in tissues.* Journal of Pharmacology and Experimental Therapeutics 127, 175-177
Hoekstra (2001)	"Immediately after venipuncture, plasma was prepared [] and stored at -80ºC"	"The amino acids [] were measured by means of high-performance liquid chromatography." (HPLC + fluorescence detection)	Fekkes et al. (1995) *Validation of the determination of amino acids in plasma by high-performance liquid chromatography using automated derivatization with o-phthaldialdehyde.* Journal of Chromatography B 669, 177-186.
Stelmasiak (1974)	"The blood was collected into heparinized tubes, centrifuged immediately and the plasma kept frozen at -20°C until it was used for determinations within 1 week."	"Tryptophan was determined using the method of Denckla and Dewey (1967) in whole plasma and also in an ultrafiltrate []." (spectrofluorometry)	Denckla and Dewey (1967) *The determination of tryptophan in plasma, liver and urine.* J. lab. clin. Med. 69, 160-169.
Sawa (1981)	"Immediately after blood collection, blood was centrifuged at 1000 g for 15 min at ice cold, and the plasma was kept frozen at -20°C till measurement."	"The contents of free (non-albumin bound) tryptophan and total tryptophan were determined spectrofluorometrically."	Denckla and Dewey (1967) *The determination of tryptophan in plasma, liver and urine.* J. lab. clin. Med. 69, 160-169.
Mokhtar (1997)	"Serum was prepared within 60-120 min from venesection and was analyzed either freshly or within 2 days of storage at -20°C."	"Free (ultra-filterable) and total (free plus albumin-bound) Trp concentrations were determined fluorimetrically as described previously (Badawy and Evans 1976)."	Badawy AA-B, Evans M (1976): *Animal liver tryptophan pyrrolases - Absence of apoenzyme and of hormonal induction mechanism from species sensitive to tryptophan toxicity.* Biochem J 158:79-88.<br><br>which uses a modified version of Denckla and Dewey (1967).
Palmio (2005)	"The samples were [] stored at -70°C before the analyses."	"[] high-performance liquid chromatography."	

The information presented in this table is selected excerpts from the reviewed studies' method sections, and not the complete method descriptions.
